# The tobacco-specific carcinogen NNK induces pulmonary tumorigenesis via nAChR/Src/STAT3-mediated activation of the renin-angiotensin system and IGF-1R signaling

**DOI:** 10.1038/s12276-023-00994-2

**Published:** 2023-06-01

**Authors:** Hye-Jin Boo, Hye-Young Min, Su Jung Hwang, Hyo-Jong Lee, Jae-Won Lee, Sei-Ryang Oh, Choon-Sik Park, Jong-Sook Park, You Mie Lee, Ho-Young Lee

**Affiliations:** 1 College of Pharmacy and Research Institute of Pharmaceutical Sciences, Seoul National University, Seoul, 08826 Republic of Korea; 2 Department of Histology, College of Medicine, Jeju National University, Jeju, 63243 Republic of Korea; 3 School of Pharmacy, Sungkyunkwan University, Suwon, 16419 Republic of Korea; 4 Natural Medicine Research Center, Korea Research Institute of Bioscience and Biotechnology, Cheongju-si, Chungcheongbuk-do 28116 Republic of Korea; 5 Soonchunhyang University Bucheon Hospital, Bucheon, Gyeonggi-do 14584 Republic of Korea; 6 Vessel-Organ Interaction Research Center (VOICE, MRC), College of Pharmacy, Kyungpook National University, Daegu, 41566 Republic of Korea

**Keywords:** Cancer, Non-small-cell lung cancer

## Abstract

The renin-angiotensin (RA) system has been implicated in lung tumorigenesis without detailed mechanistic elucidation. Here, we demonstrate that exposure to the representative tobacco-specific carcinogen nitrosamine 4-(methylnitrosamino)-1-(3-pyridyl)-1-butanone (NNK) promotes lung tumorigenesis through deregulation of the pulmonary RA system. Mechanistically, NNK binding to the nicotinic acetylcholine receptor (nAChR) induces Src-mediated signal transducer and activator of transcription 3 (STAT3) activation, resulting in transcriptional upregulation of angiotensinogen (*AGT*) and subsequent induction of the angiotensin II (AngII) receptor type 1 (AGTR1) signaling pathway. In parallel, NNK concurrently increases insulin-like growth factor 2 (IGF2) production and activation of IGF-1R/insulin receptor (IR) signaling *via* a two-step pathway involving transcriptional upregulation of *IGF2* through STAT3 activation and enhanced secretion from intracellular storage through AngII/AGTR1/PLC-intervened calcium release. NNK-mediated crosstalk between IGF-1R/IR and AGTR1 signaling promoted tumorigenic activity in lung epithelial and stromal cells. Lung tumorigenesis caused by NNK exposure or alveolar type 2 cell-specific Src activation was suppressed by heterozygous *Agt* knockout or clinically available inhibitors of the nAChR/Src or AngII/AGTR1 pathways. These results demonstrate that NNK-induced stimulation of the lung RA system leads to IGF2-mediated IGF-1R/IR signaling activation in lung epithelial and stromal cells, resulting in lung tumorigenesis in smokers.

## Lung cancer: an opportunity to repurpose available drugs

A cellular signaling pathway primarily known for modulating blood pressure also drives the progression of lung cancers arising from tobacco use. Dysfunction in the renin-angiotensin system can lead to hypertension, and many patients are treated with drugs targeting this signaling pathway. The renin-angiotensin system is also involved in certain cancers, and researchers led by Ho-Young Lee at Seoul National University, South Korea, have shown how a tobacco-derived carcinogen called NNK promotes lung cancer via this pathway. Working with cultured cells and animal models, the researchers showed that NNK stimulates production of angiotensin in respiratory tissues, resulting in IGF2 secretion. The IGF-1R signaling activation ultimately leads to transformation of pulmonary epithelial cells into cancerous ones. Antihypertensive drugs that act on the renin-angiotensin pathway suppressed NNK-mediated tumorigenesis in mice, highlighting the possibility of preventing lung cancer with existing drugs.

## Introduction

Despite advanced treatment strategies, non-small cell lung cancer (NSCLC) is the main cause of cancer-related deaths worldwide^1^. Tobacco smoking (TS) is the primary risk factor for lung cancer^2^. Among the known tobacco-specific carcinogens (TCs), 4-(methylnitrosamino)-1-(3-pyridyl)-1-butanone (NNK) is one of the most potent TCs that induce epigenetic and genetic alterations^3^. In addition to the genocentric mechanisms, NNK can stimulate various cancer-prone signaling cascades by binding to β-adrenergic receptor (β-AR) or nicotinic acetylcholine receptor (nAChR)^3–5^. Therefore, TS can induce lung tumorigenesis *via* multiple pathways.

The renin-angiotensin (RA) system, which plays a key role in blood pressure homeostasis and electrolyte balance, is a major target for the development of antihypertensive drugs^6^. Angiotensinogen (AGT) is primarily and constitutively produced in the liver, released into the systemic circulation, and converted into angiotensin I (AngI) by renin^7^. AngI is in turn converted to angiotensin II (AngII) by the activity of angiotensin converting enzyme (ACE), which is primarily distributed in pulmonary tissues^6^. AngII, the primary hormone of the RA system, binds to type 1 (AGTR1) and type 2 (AGTR2) AngII receptors^6^, of which AGTR1 mediates the majority of the pathological effects of AngII^6^. The RA system has been implicated in TS-induced pulmonary disorders and lung cancer^6–8^. AngII/AGTR1 signaling was found to increase blood flow to tumors by inducing vascular endothelial growth factor (VEGF) release^7^ and activating c-Src^7^ and insulin-like growth factor 1 receptor (IGF-1R)^9^, key molecules involved in signaling pathways for cellular transformation and survival^10^. Upregulated expression of AGTR1 and AGTR2 was observed in the lung adenomatous lesions of mice exposed to NNK^11^. Both ACE inhibitors and AGTR1 blockers (ARBs) were shown to inhibit tumor growth in animal models of breast, ovarian, lung, gastric and renal cancers^7^, and silencing AGTR2 expression suppressed the development of NNK-induced lung cancer^12^. However, there is no mechanistic explanation of how the RA system regulates TS-mediated lung tumorigenesis.

Herein, we investigated the mechanisms underlying TS-mediated control of the RA system. We identified that Src-mediated signal transducer and activator of transcription 3 (STAT3) activation resulting from NNK binding to nAChRs leads to the transcriptional upregulation of *AGT* followed by increased production of AngII. The subsequent autocrine and paracrine activation of AGTR1 leads to Ca^2+^-intervened secretion of IGF2, ultimately promoting lung tumor development *via* transformation of pulmonary epithelial cells and protumoral polarization of fibroblasts and macrophages in the tumor microenvironment. These results provide a novel concept that TS creates a tumor-prone microenvironment in the lungs through disruption of the local RA system and subsequent activation of IGF-1R/IR signaling, ultimately promoting lung tumorigenesis.

## Materials and methods

### Cell culture

BEAS-2B cells were purchased from the American Type Culture Collection (ATCC; Manassas, VA, USA). Human bronchial epithelial (HBEL)/p53i cells were kindly provided by Dr. John Minna (University of Texas Southwestern Medical Center, Dallas, TX, USA)^13^, and 1799, 1198, and 1170-I cells were kindly provided by Dr. Curtis C. Harris (National Cancer Institute, USA)^14^. These cells were cultured in K-SFM (Thermo Fisher Scientific, Waltham, MA, USA) with 5 ng/mL recombinant epidermal growth factor (EGF) and 50 μg/mL bovine pituitary extracts. MLE-12 murine alveolar epithelial cells, THP-1 human monocytes/macrophages, and Wi38 human lung fibroblasts were purchased from ATCC. MLE-12 cells were cultured in HITES medium [DMEM-F12 media (Welgene, Inc., Gyeongssan-si, Republic of Korea) containing 1× insulin-transferrin-selenium solution (Thermo Fisher Scientific), 10 nM hydrocortisone, 10 nM β-estradiol, 10 mM HEPES, and 2 mM L-glutamine (Welgene)] with 2% fetal bovine serum (FBS) (Welgene) and 1× antibiotic-antimycotic solution (100 units/mL penicillin, 10 mg/mL streptomycin sulfate, and 25 μg/mL amphotericin B in 0.85% NaCl solution; Welgene). THP-1 and Wi38 cells were cultured in RPMI 1640 medium and DMEM, respectively, with 10% FBS and 1× antibiotic-antimycotic solution. Cells were maintained at 37 °C in a humidified atmosphere with 5% CO_2_.

### Reagents

Dimethyl sulfoxide (DMSO), methyllycaconitine (MLA), an anti-α-smooth muscle actin (α-SMA) antibody, and the other chemicals used in the study were purchased from Sigma‒Aldrich (St. Louis, MO, USA), unless otherwise indicated. Propranolol, atenolol, ICI-118,551, and mecamylamine (MCA) were purchased from Tocris Bioscience (Bristol, UK), and NNK was purchased from Toronto Research Chemicals (TRC; Toronto, ON, Canada). Antibodies against phosphorylated IGF-1R (pIGF-1R) (Y1131 and Y1135/36), pSTAT3 (Y705), pSrc (Y416), pAkt (S473), pp44/p42 MAPK (pERK1/2; T202/Y204), ERK1/2, Akt, Src, STAT3, IGF-1R, β-tubulin (Tubulin), and arginase 1 (Arg1) were purchased from Cell Signaling Technology (Danvers, MA, USA). The anti-actin, anti-IGF-1R, and anti-IGF1 antibodies were purchased from Santa Cruz Biotechnology (Dallas, TX, USA). Antibodies against proliferating cell nuclear antigen (PCNA), IGF2, Ki67, and AGT were purchased from Abcam (Cambridge, UK). Anti-FAK and anti-pFAK (Y861) antibodies were acquired from BD Biosciences (San Jose, CA, USA) and Thermo Fisher Scientific, respectively.

### Cell viability, anchorage-dependent and anchorage-independent colony formation, and foci formation assays

The suppression of NNK-induced transformed phenotypes, including cell viability, anchorage-dependent colony formation, and formation of foci, following inhibition of the RA system was evaluated as previously described^15^.

### Western blot analysis

The cells were starved with supplement-free K-SFM and subsequently stimulated with NNK or AngII. When necessary, the cells were pretreated with inhibitors 3 h before stimulation with NNK or AngII. Western blot analysis was performed as described previously^15^.

### Transfection experiments

For the transient knockdown of α7nAChR, SRC, AGT, and STAT3, the cells were transfected with scrambled or gene-specific small interfering RNAs (siRNAs, purchased from Bioneer (Daejeon, Republic of Korea)) using the JetPRIME transfection reagent (Polyplus-transfection SA, Illkirch, France) according to the manufacturer’s instructions. Gene knockdown was confirmed with Western blotting or real-time polymerase chain reaction (PCR). For generation of stable knockdown cell lines with reduced AGTR1 or IGF2R expression, 1170-I, 1799, or BEAS-2B cells were transduced with lentiviral particles with shRNA clones against AGTR1 or IGF2R (Sigma‒Aldrich), followed by selection with puromycin.

### Cloning of AGT promoter construct and luciferase reporter assay

The promoter sequence of *AGT* (−306/+36) was retrieved as previously described^16^. The putative STAT3 binding sites in the promoter sequence of *AGT* were determined using the PROMO bioinformatics web server^17^. The PCR products of the *AGT* promoter were generated using cDNA from BEAS-2B cells. The *AGT* promoter construct was cloned by ligating the PCR products to the pGL3-basic vector at the Nhel/HindIII restriction sites. Mutations were introduced at the STAT3 binding site of the *AGT* promoter using a QuikChange II Site-Directed Mutagenesis Kit (Agilent, Santa Clara, CA, USA).

Activity of the *AGT* promoter was measured with a reporter gene assay performed using a luciferase assay system (Promega Corp., Madison, WI, USA) according to the manufacturer’s protocol. Briefly, the cells were transfected with the luciferase vector containing the promoter sequence of *AGT* (pGL3-AGT) or the empty vector (pGL3) along with pSV-β-galactosidase. The cells were subsequently harvested with passive lysis buffer, and the luciferase activity was monitored using a microplate luminometer (Berthold Technologies GmbH & Co. KG, Germany). β-gal was used as the control for normalizing the transfection efficiency, and its activity was measured using a β-gal enzyme assay system (Promega).

### In silico analysis

To analyze the expression of the components of the RA system in the airway epithelium and pulmonary tumors, we used publicly available datasets deposited in the Gene Expression Omnibus (GEO) database (National Center for Biotechnology Information). Raw data pertaining to the gene expression levels and clinical information on individual patient samples were manually downloaded and analyzed using GraphPad Prism 9 (GraphPad Software, Inc., San Diego, CA, USA). Detailed procedures are described in our previous report^18^. For a heatmap, data were normalized by calculating z scores, and then, the median value of the normalized data was used. The probes used to obtain the gene expression values from each dataset are listed in Supplementary Table 1.

### Reverse transcription-PCR (RT‒PCR)

RT‒PCR and real-time PCR analyses were performed as previously described^15,18^. The relative mRNA expression was quantified using the comparative cycle threshold (CT) method, as previously described^19^. The primer sequences used for RT‒PCR are listed in Supplementary Table 2.

### Time-lapse live cell imaging

Time-lapse imaging was performed with the Operetta high-content screening system (PerkinElmer, Waltham, MA, USA) as described previously^15^.

### Animal experiments

The protocols used for animal experiments were approved by the Institutional Animal Care and Use Committee of Seoul National University. Wild-type or heterozygous *Agt* knockout (KO) (Agt^+/−^) mice were randomly grouped and treated with 3 μmol of NNK twice a week for 20 weeks. As C57BL/6 mice are highly resistant to chemical carcinogens^20^, heterozygous *Agt* KO mice with a C57BL/6 background were backcrossed for more than 8 generations into an FVB/N background. We used both male and female mice, and the number of mice of each sex was equal in each group.

We generated mice expressing lung-specific *SRC* transgenes (Src^Tg/+^ mice) under the control of the human surfactant protein C *(SFTPC*) promoter in an FVB/N. The kbpA vector downstream of *the SFTPC* promoter was kindly provided by Dr. Francesco DeMayo (Baylor College of Medicine, Houston, TX, USA). The *SFTPC*-SRC plasmid was constructed by subcloning human SRC cDNA into the *SFTPC*-kbpA vector. The plasmid was digested and subjected to gel electrophoresis to isolate the *SFTPC*-SRC transgene, which was microinjected into hybrid C3H/C57BL6 fertilized mouse eggs. Mice containing this transgene were confirmed by PCR analysis and backcrossed into an FVB/N background for over 8 generations. We used both male and female mice, and the number of mice of each sex was equal in each group.

Additionally, 2–4 weeks prior to drug administration, 5-week-old female A/J mice were randomly grouped and administered 3 μmol of NNK (dissolved in sterile PBS) by oral gavage twice a week. The drugs (1 mg/kg methyllycaconitine, 10 mg/kg dasatinib, 5 mg/kg captopril, and 25 mg/kg losartan) were administered by oral gavage for an additional 20 weeks, with or without NNK. All the drugs were dissolved in PBS before administration, with the exception of dasatinib. Dasatinib powder was dissolved in an 80 mM citric acid solution, or a 100 mg dasatinib tablet was suspended in sterile PBS (100 μL). The mice were euthanized prior to gross evaluation of the lung tumors. Microscopic evaluation of lung tumors was performed as described previously^15^.

For cigarette smoke (CS) exposure, 8-week-old male and female FVB/N mice were whole-body-exposed to room air (RA) or CS for 28 days (*n* = 8–12 mice per group) using a smoking machine (SciTech Korea, Inc., Seoul, Korea) as previously described^21^. Cigarette smoke was generated from 3R4F Research Cigarettes, containing 11.0 mg of total particulate matter, 9.4 mg of tar, and 0.76 mg of nicotine per cigarette (Tobacco and Health Research Institute, University of Kentucky, Lexington, KY, USA).

### Bronchoalveolar lavage fluid (BALF) preparation

Bronchoalveolar lavage fluid (BALF) samples were collected as described previously^22^. Briefly, lungs were collected from vehicle- and NNK-treated (for 20 weeks) mice. The trachea was cannulated with a 20-gauge catheter. The lungs were lavaged four times with 0.75 mL of cold sterile PBS containing protease inhibitors by syringe. The BALF samples were centrifuged at 800 x *g* for 10 min at 4 °C, and then, BALF supernatants were stored at −80 °C until analysis.

### Angiotensin II ELISA

The level of angiotensin II in murine BALFs and serum was determined by an enzyme-linked immunosorbent assay (ELISA) using an angiotensin II ELISA kit (Cloud-Clone Corp., Katy, TX, USA) according to the manufacturer’s instructions.

### Immunofluorescence (IF) and immunohistochemistry studies

IF and IHC analyses were performed to detect the expression of phosphorylated IGF-1R (pIGF-1R, Y1131 or Y1135/36), pSrc (Y416), Src, AGT, pSTAT3 (Y705), Arg1, α-SMA, Ki67, and PCNA in the tumors, as previously described^15^.

### Statistical analysis

Data are presented as the mean ± standard deviation (SD). All in vitro experiments were independently performed at least twice, and the representative result is presented herein. Statistical significance was analyzed using GraphPad Prism 9 (GraphPad Software, Inc., La Jolla, CA, USA). Detailed statistical analysis methods are described in each figure legend. *p-*values of <0.05 were considered to be statistically significant.

## Results

### AGT expression is upregulated in the airway epithelium and lung tumors of smokers, and AngII increases the protumoral activities of airway epithelial cells

**Fig. 1:**
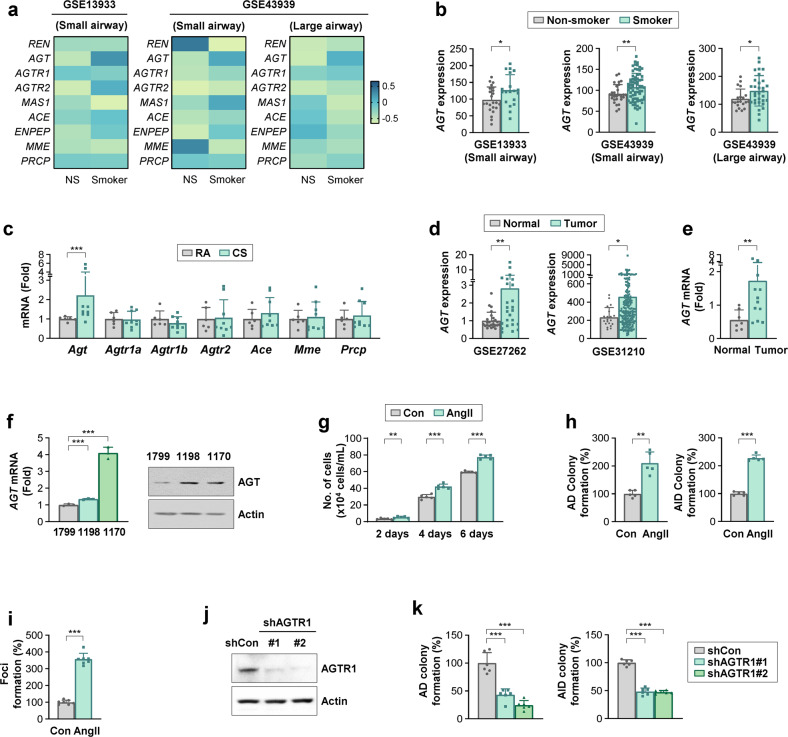
Increase in AGT expression in the airway epithelium of smokers and lung tumors. **a**, **b**, and **d** Analysis of publicly available datasets for the expression of the RA system components in airway epithelium of smokers [GSE13933: *n* = 19; GSE43939 (small airway): *n* = 69; GSE43939 (large airway): *n* = 31] vs. nonsmokers [GSE13933: *n* = 23; GSE43939 (small airway): *n* = 28; GSE43939 (large airway): *n* = 20] (**a**, **b**) and *AGT* expression in tumors (GSE27262: *n* = 25; GSE31210: *n* = 204) vs. normal (GSE27262: *n* = 25; GSE31210: *n* = 20) lung tissues (**d**). The median value of the normalized data is depicted as a heatmap (**a**). **c**, **e** Real-time PCR analyses for the level of RA system components in murine lung tissues of room air (RA)- and cigarette smoking (CS)-exposed mice (RA: *n* = 8; CS: *n* = 12) (**c**) and *AGT* mRNA expression in patient-derived lung tumors (*n* = 12) and adjacent normal lung tissues (*n* = 7) (**e**). **f** Real-time PCR and Western blot (WB) analyses for AGT expression in 1799, 1198, and 1170 cells. **g–i** Regulation of proliferation (**g**) and anchorage-dependent (AD) and anchorage-independent (AID) colony formation (**h**) of BEAS-2B cells and formation of foci (**i**) of HBEL/p53i cells by treatment with 0.1 μM angiotensin II (AngII). **j** WB analysis showing the AGTR1 expression level in 1170 cells stably transfected with the control (1170/shCon) or AGTR1 (1170/shAGTR1) shRNAs. **k** AD and AID colony formation of 1170/shCon and 1170/shAGTR1 cells. The bars represent the mean ± SD; *p < 0.05, **p < 0.01, and ***p < 0.001, as determined by Mann‒Whitney test (**b**, **c**, **d**, and **e**), Wilcoxon signed rank test (**d**, left), two-tailed Student’s *t*-test (**g**, **h**), two-tailed Welch’s *t*-test (**h**, **i**) by comparison with the indicated group, or one-way ANOVA with Dunnett’s post hoc test (**f**, **k**).

To determine the role of the RA system in TS-induced lung tumorigenesis, we first analyzed the GEO database for the expression of RA system components^23^, including *AGTR1* (encoding AT_1_R), *AGTR2* (encoding AT_2_R), *ACE* (encoding angiotensin-converting enzyme), *ENPEP* (encoding aminopeptidase N), *MME* (encoding neprilysin), and *PRCP* (encoding prolylcarboxypeptidase), in the airway epithelium (Fig. 1a). Two datasets consistently showed significant upregulation of *AGT* expression in the airway epithelium of smokers compared to that in nonsmokers (Fig. 1a, b). However, the expression of other RA system components showed minimal and inconsistent changes across these datasets (Supplementary Fig. 1a). *Agt* mRNA expression was also significantly increased in TS-exposed murine lungs (Fig. 1c). Analysis of two different public datasets (Fig. 1d) and our validation experiments using human lung tissues (Fig. 1e) further revealed significantly increased mRNA levels of *AGT* in lung tumors compared with normal lung tissues. These findings suggest that *AGT* expression contributes to TS-induced lung tumorigenesis.

We next analyzed the in vitro lung carcinogenesis system, which comprises BEAS-2B human bronchial epithelial (HBEL) cell derivatives established in vivo by chronic exposure to vehicle (1799) or cigarette smoke condensate (CSC) (1198 and 1170)^14^. Real-time PCR and Western blot (WB) analyses revealed obvious increases in AGT mRNA and protein expression in premalignant (1198) and malignant (1170) cells^14^ compared with their control cells (1799) (Fig. 1f). Analysis of the direct effects of AngII on the transformation of pulmonary epithelial cells revealed that AngII exposure significantly increased proliferation (Fig. 1g), colony formation in anchorage-dependent (AD) and anchorage-independent (AID) culture conditions (Fig. 1h), and formation of foci (Fig. 1i). In addition, 1170 cells, in which AGTR1 expression was ablated through stable transfection with shRNAs (Fig. 1j), showed significant ablation in transformed phenotypes, including colony formation under the AD and AID culture conditions (Fig. 1k). Treatment with NNK significantly increased the anchorage-dependent (AD) and anchorage-independent (AID) colony-forming capacities of 1170 cells. These NNK-induced transformed phenotypes were blunted in 1170 cells, in which AGTR1 expression was silenced. These results indicate the role of AGTR1 in NNK-induced acquisition of transformed phenotypes in lung epithelial cells (Supplementary Fig. 1b). These findings collectively suggest that TS-mediated transcriptional upregulation of *AGT* induces transformation of airway epithelial cells.

### AGT expression promotes NNK-mediated lung tumorigenesis

**Fig. 2:**
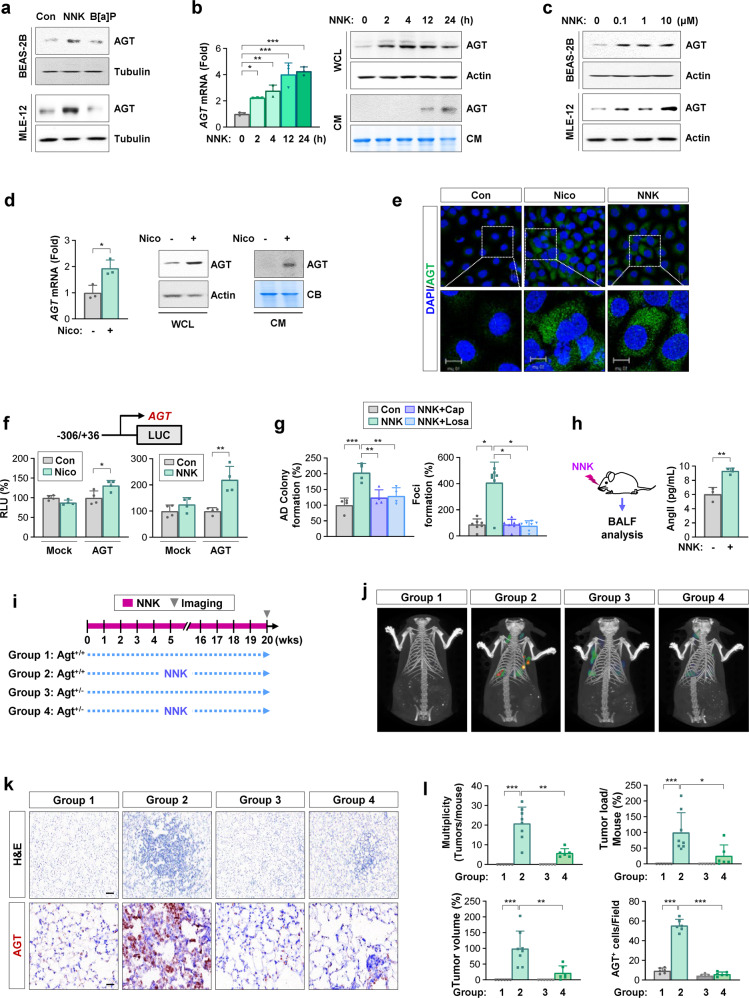
Role of AGT in NNK-induced lung tumorigenesis. **a–d** Western blot (WB) analysis of whole cell lysates (WCL) (**a–d**) or conditioned medium (CM) (**b**, **d**) and real-time PCR (**b**, **d**) analysis for the regulation of *AGT* expression in BEAS-2B (**a–d**) and MLE-12 (**a**, **c**) lung epithelial cells treated with 10 μM (**a**, **b**) or the indicated concentrations (**c**) of NNK, benzo[a]pyrene (B[a]P, 10 μM; **a**), or nicotine (Nico, 10 μM) (**d**) for 24 h. **e**, **f** Immunofluorescence staining for the regulation of AGT expression (**e**) and reporter analysis of *AGT* promoter activity (**f**) in BEAS-2B cells treated with nicotine (10 μM) or NNK (10 μM) for 24 h. **g** Anchorage-dependent (AD) colony formation of BEAS-2B cells (left) and formation of foci of HBEL/p53i cells (right) after treatment with NNK (10 μM), either alone or together with captopril (Cap, 1 μM) and losartan (Losa, 10 μM) for 2 weeks. **h** ELISAs of AngII levels in the bronchoalveolar lavage fluid (BALF) from the lungs of the mice exposed to vehicle (PBS) or NNK (oral gavage, 3 μmol). **i** Schematic diagram of the experimental procedure. **j**, **l** Regulation of NNK-induced lung tumor formation in the Agt^+/+^ and Agt^+/−^ mice treated with vehicle (PBS) or NNK (oral gavage, 3 μmol), as determined by IVIS Spectrum CT imaging using an MMP680 sense probe (**j**) and microscopic analysis using H&E-stained tissues (Group 1, 3, 4: *n* = 6; Group 2: *n* = 8) (**l**). **k** Immunohistochemical analysis of the regulation of AGT in the lungs of the vehicle- or NNK-treated Agt^+/+^ and Agt^+/−^ mice (*n* = 6). The bars represent the mean ± SD; *p < 0.05, **p < 0.01, and ***p < 0.001, as determined by one-way ANOVA with Tukey’s post hoc test (**b**, **g**, **l**), a two-tailed Student’s *t*-test (**d**, **f**, **h**) by comparison with the indicated group, or Kruskal‒Wallis test with Dunn’s post hoc test (**l**). Scale bars: 20 μm (**e**); 10 μm (**e**, insets); 25 μm (**k**, H&E images); 100 μm (**k**, IHC images). Con: control.

To identify the mechanisms underlying TS-mediated *AGT* expression, we determined the effect of two representative TCs, NNK and benzo[a]pyrene (B[a]P), on *AGT* expression in lung epithelial cell lines. Exposure to NNK, but not B[a]P, markedly increased AGT protein expression in bronchial (BEAS-2B) and alveolar (MLE-12) epithelial cells (Fig. 2a). Real-time PCR and WB analyses further revealed that NNK induced time-dependent increases in *AGT* transcription in BEAS-2B cells as early as 2 h after exposure, which were followed by AGT secretion into conditioned medium (CM) (Fig. 2b). Notably, NNK increased AGT expression in BEAS-2B and MLE-12 cells at doses as low as 0.1 μM (Fig. 2c), which is physiologically relevant, as active smokers have shown 2 × 10^−4^ μM NNK as an average steady-state serum concentration, which can acutely increase to 10-100 μM in serum or to 1 mM at the mucosal surface immediately after smoking^24^. Nicotine, a precursor of NNK, also increased *AGT* transcription and AGT secretion (Fig. 2d). Immunofluorescence (IF) analysis further confirmed the nicotine- and NNK-induced AGT expression (Fig. 2e). Luciferase reporter assays using a reporter vector carrying the *AGT* promoter (from −306 to +36)^16^ revealed that treatment with NNK or nicotine significantly stimulated *AGT* promoter activity in BEAS-2B cells (Fig. 2f). Moreover, exposure to NNK significantly increased AD colony formation (Fig. 2g, left) in BEAS-2B cells and formation of foci (Fig. 2g, right) in HBEL cells with loss of p53 (HBEL/p53i)^13^, which were significantly abrogated by treatment with an ACE inhibitor (captopril) or an AGTR1 antagonist (losartan). Because captopril and losartan inhibited the NNK-mediated effects, we additionally determined the expression of renin and AGT and their modulation by NNK treatment in BEAS-2B and HBEL/p53i cells. A previous report suggested determining gene expression levels based on Ct values (high expression: Ct < 20; low expression: Ct > 30; no expression: Ct > 35)^25^. We found that the Ct values of the renin and ACE genes were ~30 and that the expression levels of these genes were significantly increased by treatment with NNK (Supplementary Fig. 1c). These findings suggest that BEAS-2B and HBEL/p53i lung epithelial cells express renin and ACE at low levels and that the expression of these genes was highly upregulated by NNK exposure. These results also suggest that NNK causes local RA system activation in pulmonary epithelial cells through transcriptional upregulation of *AGT*.

We analyzed the involvement of AGT in NNK-induced pulmonary tumorigenesis. We first confirmed increased AngII levels in the bronchoalveolar lavage fluid (BALF) from NNK-treated mice for 20 weeks (Fig. 2h). Wild-type (Agt^+/+^) and heterozygous *Agt* knockout (Agt^+/-^) mice were administered NNK for 20 weeks, and pulmonary tumor formation was subsequently analyzed (Fig. 2i). IVIS imaging using an MMP680 sense probe (Fig. 2j) and microscopic evaluation of the H&E-stained lung tissues (Fig. 2k, l) revealed that exposure to NNK significantly increased the multiplicity, volume, and load of lung tumor nodules, and these changes were significantly suppressed in the Agt^+/−^ mice. IHC analysis of lung tissues confirmed an increase in AGT in lung tissues from the NNK-exposed mice, which was significantly attenuated in the Agt^+/−^ mice (Fig. 2k, l). To determine the cell types that express AGT upon NNK exposure, we examined AGT expression in mucin1-positive (Muc1^+^) alveolar type 2 epithelial cells (AT2s) and F4/80^+^ macrophages, the most abundant immune cell type in the lungs^26^. We observed high levels of AGT expression in both Muc1^+^ AT2s and F4/80^+^ macrophages in the lungs of the NNK-treated Agt^+/+^ mice but not in those of the Agt^+/−^ mice (Supplementary Fig. 2).

Given the systemic impact of NNK administered by oral gavage, we additionally assessed the levels of angiotensinogen (*Agt*) and renin (*Ren*) expression in the lung, *Agt* expression in the liver, renin expression in the kidney, and circulating levels of angiotensin II (AngII) in the mice that received oral administration of NNK. We observed significant transcriptional increases in *Ren* and *Agt* expression in the lungs of the NNK-treated mice compared with those in the vehicle-treated control group. The NNK-exposed mice showed slightly decreased *Agt* expression in the liver along with significantly increased *Ren* expression in the kidney compared with the vehicle-treated mice (Supplementary Fig. 3a). Regardless of these changes in the liver and kidney, the circulating AngII levels were minimally changed by NNK treatment (Supplementary Fig. 3b). These findings suggest that NNK exposure had major impacts on the local renin-angiotensin system. These findings collectively suggest that deregulation of the lung RA system through transcriptional upregulation of *AGT* contributes to NNK-mediated pulmonary tumorigenesis.

### c-Src mediates a transcriptional increase in *AGT* expression in lung epithelial cells and NNK-induced lung tumorigenesis

**Fig. 3:**
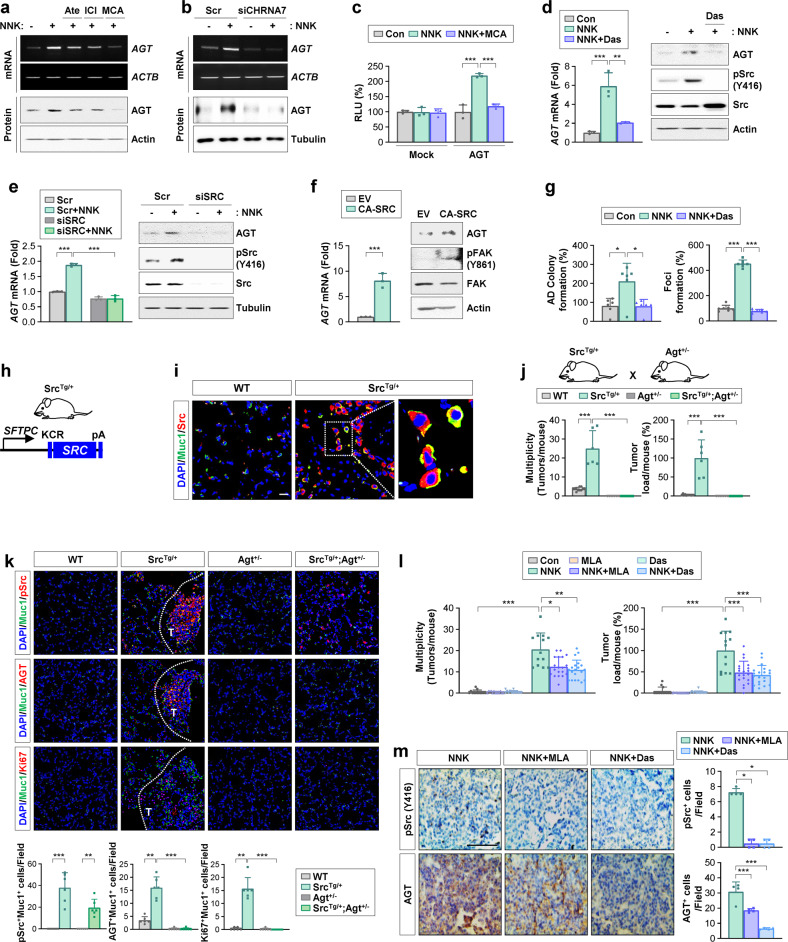
AGT plays a key role in c-Src-mediated lung tumor development. **a**, **b** RT‒PCR and Western blot (WB) analyses of BEAS-2B cells treated with NNK (10 μM), either alone or together with atenolol (Ate, 10 μM), ICI-118,551 (ICI, 10 μM), and mecamylamine (MCA, 10 μM) for 24 h (**a**), and those transfected with α7nAChR siRNAs (siCHRNA7) (**b**). **c** Luciferase reporter assay showing *AGT* promoter activity in BEAS-2B cells treated with NNK (10 μM), either alone or together with mecamylamine (MCA, 10 μM) for 24 h. **d**–**f** Real-time PCR and WB analyses of BEAS-2B cells, in which SRC was inactivated by treatment with dasatinib (Das, 0.1 μM) for 24 h (**d**) or transfection with siRNA (**e**), and those stably transfected with constitutively activated SRC (CA-SRC) (**f**). **g** Anchorage-dependent (AD) colony formation of BEAS-2B cells (left) and formation of foci of HBEL/p53i cells (right) treated with NNK (10 μM), either alone or together with dasatinib (Das, 0.1 μM) for 2 weeks. **h** Schematic diagram of the construction of Src^Tg/+^ mice. **i** Immunofluorescence (IF) analysis of the regulation of Src expression in the lungs of Src^Tg/+^ mice compared with those of wild-type (WT) mice. **j** Microscopic examination of H&E-stained lung tissues from wild-type (WT), Src^Tg/+^, Agt^+/−^, and Src^Tg/+^;Agt^+/−^ mice (*n* = 6/group) to determine tumor multiplicity and load. **k** IF staining and quantitative analyses for pSrc (pSrc at Y416), AGT, and Ki67 in Muc1^+^ alveolar type 2 epithelial cells in the lungs. **l**, **m** Mice were treated with NNK (oral gavage, 3 μmol) in the absence or presence of methyllycaconitine (MLA, oral gavage, 1 mg/kg) or dasatinib (Das, oral gavage, 10 mg/kg). **l** Microscope analyses of H&E-stained lung tissues for lung tumor formation (*n* ≥ 5). **m** Immunohistochemistry analyses of the indicated proteins in the lungs from the mice (*n* = 4). The bars represent the mean ± SD; *p < 0.05, **p < 0.01, and ***p < 0.001, as determined by one-way ANOVA with Dunnett’s post hoc test (**c**–**e**, **g**, **l**, **m**), a two-tailed Student’s t-test by comparison with the indicated group (**f**), a Kruskal‒Wallis test with Dunn’s post hoc test (**g**, **j**, **k**), or a Brown-Forsythe and Welch ANOVA test with Dunnett’s T3 post-hoc test (**k**, **l**). Scale bars: 20 μm (**i**, **k**), 50 μm (**m**).

We investigated the signal transduction pathways that were involved in NNK-induced AGT expression and the subsequent transformation of pulmonary epithelial cells. Since NNK is known to bind nAChR and β-AR^4,5^, we first assessed whether NNK binding to nAChR or β-AR leads to upregulation of AGT. We found that treatment with β-AR antagonists [atenolol (β1-AR selective) or ICI-118,551 (β2-AR selective)] had modest effects on NNK-induced AGT expression (Fig. 3a). However, treatment with a nonselective nAChR antagonist (mecamylamine; MCA) or silencing of α7nAChR expression by siRNA transfection markedly inhibited NNK-induced AGT expression (Fig. 3a, b). We further confirmed the suppression of *AGT* promoter (from −306 to +36) activity in BEAS-2B cells by mecamylamine treatment (Fig. 3c), suggesting the involvement of α7nAChR in NNK-induced AGT expression.

Since Src is a downstream component of the nAChR-mediated signaling pathway^27,28^, we hypothesized that Src has a role in NNK-induced AGT expression in lung epithelial cells. We found that inactivation of Src by treatment with dasatinib (Fig. 3d) or siRNA-mediated silencing (Fig. 3e) abrogated NNK-induced AGT upregulation. Moreover, ectopic overexpression of constitutively active Src (CA-SRC) significantly increased the mRNA and protein expression of AGT in BEAS-2B cells (Fig. 3f), and NNK-induced increases in AD colony formation (Fig. 3g) and formation of foci (Fig. 3g) were significantly suppressed by treatment with dasatinib. We confirmed the decrease in Src phosphorylation by treatment with dasatinib (Fig. 3d), siRNA-mediated silencing of Src expression (Fig. 3e), and an increase in Src-specific phosphorylation of focal adhesion kinase (FAK, Y861)^29^ by overexpression of CA-SRC (Fig. 3f) in BEAS-2B cells. These results suggest that Src mediates NNK-induced AGT upregulation and transformation of lung epithelial cells.

We further investigated whether activation of the lung RA system through NNK-induced AGT expression contributed to pulmonary tumorigenesis through Src activation. A previous report showed that AT2s are putative cell-of-origin for lung adenocarcinoma in both humans and rodents^30^. Hence, we established a transgenic murine model with lung-specific c-SRC expression (Src^Tg/+^ mouse) under the control of the surfactant protein C (*SFTPC*) promoter, which specifically targets gene expression in AT2s in the lung^31^ (Fig. 3h). IF analysis revealed elevated Src expression in Muc1^+^AT2s of the Src^Tg/+^ mice compared with those of the WT mice (Fig. 3i). To assess whether changes in AGT expression influence lung tumor development induced by Src activation, we crossed Src^Tg/+^ mice with Agt^+/-^ mice^32^. Quantitative analysis of the lungs from four different groups of mice (WT, Src^Tg/+^, Agt^+/−^, and Src^Tg/+^;Agt^+/−^) showed that the Src^Tg/+^;Agt^+/−^mice had a significantly reduced multiplicity and load of spontaneous lung tumors compared with the Src^Tg/+^ mice (Fig. 3j). Furthermore, IF analysis revealed that the expression of pSrc, AGT, and the proliferation marker Ki67 in Muc1^+^AT2s of the Src^Tg/+^;Agt^+/−^ mice was significantly lower than that in the Src^Tg/+^ mice (Fig. 3k). Moreover, pharmacological inhibition of AChR or Src by treatment with methyllycaconitine (MLA) or dasatinib, respectively, significantly suppressed the NNK-induced tumor multiplicity and load in mice (Fig. 3l), along with decreases in the expression of pSrc and AGT (Fig. 3m). These findings collectively suggest that AGT expression contributes to Src-mediated lung epithelial cell transformation and lung tumorigenesis.

### STAT3 activated by NNK-induced nAChR/Src signaling stimulates *AGT* transcription, leading to transformation of pulmonary epithelial cells

**Fig. 4:**
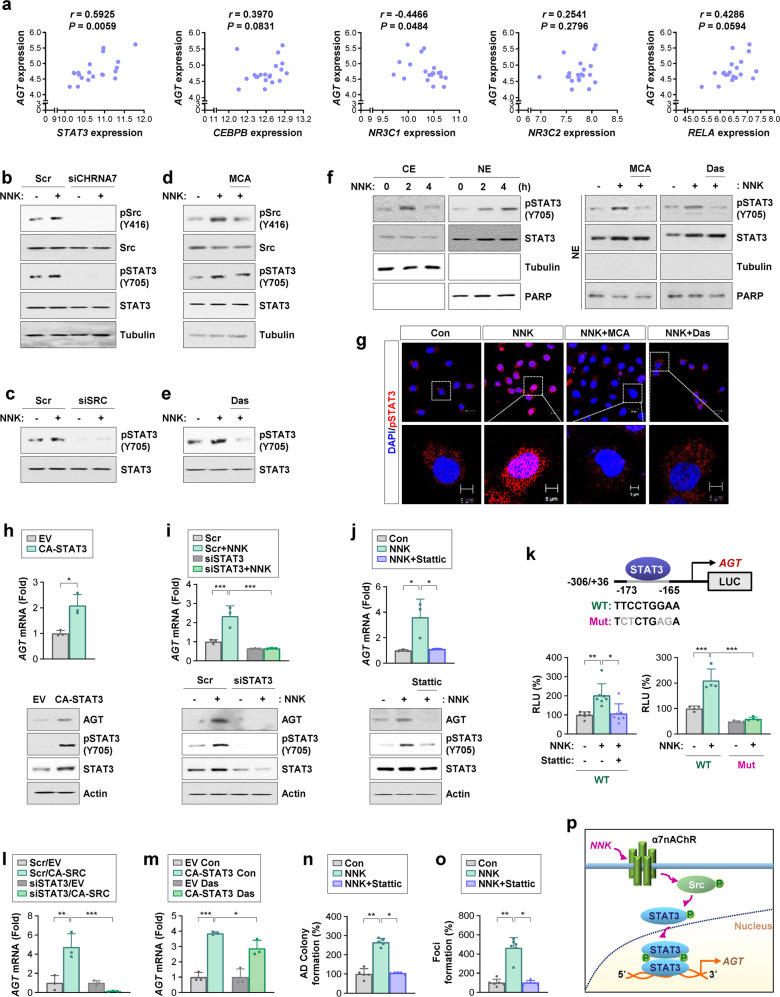
NNK-mediated activation of the α7nAChR/Src/STAT3 pathway upregulates *AGT* transcription, leading to transformation of pulmonary epithelial cells. **a** Analysis of the GSE37768 dataset (*n* = 20) for the Spearman rank correlation between *AGT* mRNA expression and mRNA expression of several transcription factors, such as *STAT3*, *CEBPB*, *NR3C1*, *NR3C2*, and *RELA*. In the GSE37768 dataset, data from peripheral lung tissues without COPD (*n* = 20) were used for correlation analysis. **b–f** Western blot (WB) analysis of whole cell lysates (**b**–**e**) and nuclear and cytosolic extracts (NE vs. CE) (**f**) for the indicated proteins in BEAS-2B cells that experienced NNK (10 μM) exposure, either alone or together with siRNA-mediated silencing of α7nAChR (**b**) or Src (**c**) expression or pharmacological blockade of α7nAChR or Src by treatment with mecamylamine (MCA, 10 μM) (**d**, **f**) or dasatinib (Das, 0.1 μM) (**e**, **f**) for 4 or 24 h. **g** Immunofluorescence staining of pSTAT3 in BEAS-2B cells treated with NNK (10 μM), either alone or together with mecamylamine (MCA, 10 μM) or dasatinib (Das, 0.1 μM) for 4 h. Scale bars: 20 μm; 5 μm (insets). **h**–**j** Real-time PCR and WB analyses of BEAS-2B cells transfected with constitutively activated STAT3 (CA-STAT3) (**h**) or those treated with NNK (10 μM), either alone or together with siRNA-mediated STAT3 silencing (**i**) or with treatment with Stattic (1 μM) for 24 h (**j**). **k** Luciferase reporter assay for the activity of the *AGT* promoter, either wild-type (WT) or carrying a mutation in the putative STAT3 binding site, in BEAS-2B cells treated with NNK (10 μM) in the absence or presence of Stattic (1 μM) for 24 h. **l**, **m** Real-time PCR analysis of BEAS-2B cells transfected with constitutively active SRC (CA-SRC) (**l**) or CA-STAT3 (**m**) followed by transfection with STAT3-targeting siRNA (**l**) or treatment with dasatinib (Das, 0.1 μM) for 24 h (**m**). **n**, **o** Regulation of NNK-mediated anchorage-dependent (AD) colony formation of BEAS-2B cells (**n**) and formation of foci of HBEL/p53i cells (**o**) by treatment with Stattic (1 μM) for 2 weeks. **p** Schematic diagram of the regulation of NNK-mediated AGT expression through the α7nAChR/Src/STAT3 pathway. The bars represent the mean ± SD; *p < 0.05, **p < 0.01, and ***p < 0.001, as determined by a two-tailed Student’s t-test by comparison with the indicated group (h), one-way ANOVA with Dunnett’s post hoc test (i-m), or Kruskal‒Wallis test with Dunn’s post hoc test (**k, n, o**).

We examined the transcription factors responsible for NNK-induced *AGT* expression and subsequent transformation of pulmonary epithelial cells. Since several transcription factors, such as STAT3, CEBPB, NR3C1, NR3C2, and NF-κB p65 (RelA), have been suggested to regulate AGT expression^33–35^, we analyzed two publicly available datasets (GSE18385 and GSE37768) to investigate whether these factors are involved in NNK-mediated AGT expression. We found that only *STAT3* expression commonly showed a significant correlation with *AGT* expression in the two datasets (Fig. 4a, Supplementary Fig. 4). Thus, we hypothesized that STAT3, a downstream effector of the nAChR/Src-mediated signaling pathway^27,28^, is involved in NNK-induced *AGT* transcription. Indeed, while exposure to NNK induced Src and STAT3 phosphorylation in BEAS-2B cells, transfection with α7nAChR- (Fig. 4b) or Src- (Fig. 4c) specific siRNAs or treatment with mecamylamine (MCA) (Fig. 4d) or dasatinib (Das) (Fig. 4e) suppressed the NNK-induced Src and STAT3 activation. WB (Fig. 4f) and IF (Fig. 4g) analyses further confirmed that NNK induced time-dependent increases in phosphorylated STAT3 in the nucleus, which were suppressed by treatment with mecamylamine or dasatinib. Moreover, overexpression of constitutively active STAT3 (CA-STAT3) upregulated the mRNA and protein expression of AGT in BEAS-2B cells (Fig. 4h). In contrast, siRNA-mediated STAT3 silencing (Fig. 4i) or treatment with Stattic (Fig. 4j) abrogated the NNK-induced *AGT* transcription. We then assessed potential STAT3 binding sites in the *AGT* promoter using a publicly available bioinformatics web server and found two potential STAT3 binding sites (−173 to −165) in the *AGT* promoter (from -306 to +36)^16^. The luciferase reporter assay revealed that treatment with Stattic or mutations in the potential STAT3 binding site significantly attenuated NNK-induced *AGT* promoter activity (Fig. 4k).

Notably, constitutive Src activation failed to induce *AGT* transcription in BEAS-2B cells, in which STAT3 had been silenced by siRNA transfection (Fig. 4l). In contrast, inactivation of Src by treatment with dasatinib minimally affected *AGT* transcription in BEAS-2B cells, in which activation of STAT3 was achieved by transfection with constitutively active STAT3 (Fig. 4m). The NNK-induced acquisition of transformed phenotypes in pulmonary epithelial cells, including AD colony formation (Fig. 4n) and formation of foci (Fig. 4o), was significantly abolished by treatment with Stattic. These results suggest that the NNK-induced activation of α7AChR/Src signaling results in the STAT3-mediated transcriptional increase in *AGT* expression, causing transformation of pulmonary epithelial cells (Fig. 4p).

### The AngII/AGTR1-induced increase in intracellular calcium stimulates IGF2 secretion and IGF-1R activation in pulmonary epithelial cells and macrophages, promoting NNK-mediated lung tumorigenesis

**Fig. 5:**
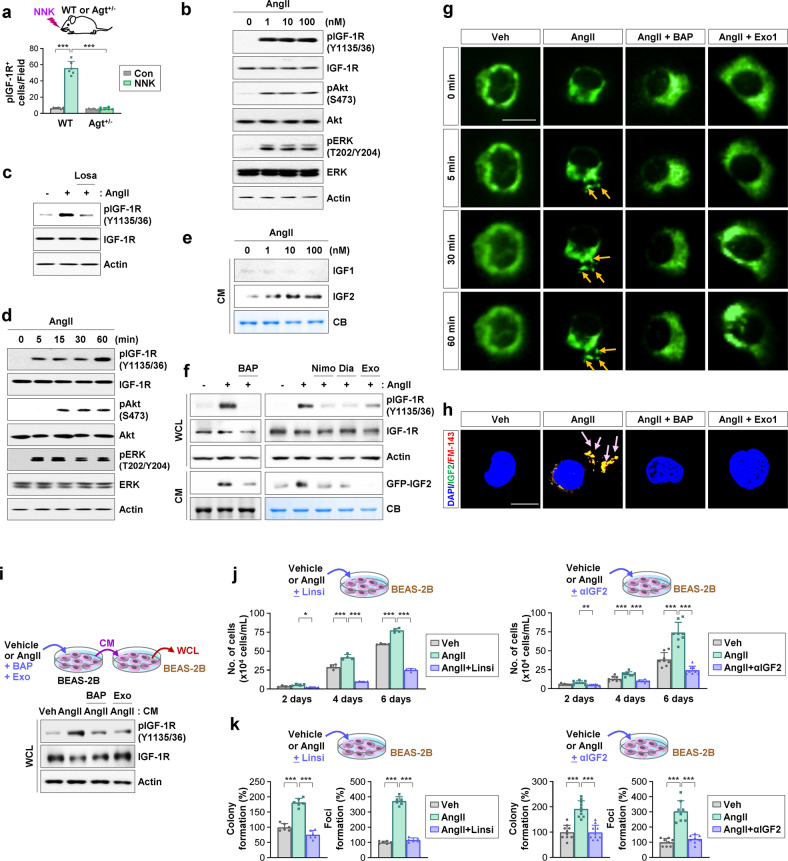
AngII/AGTR1-mediated calcium release stimulates IGF2 secretion and IGF-1R signaling activation in pulmonary epithelial cells, promoting NNK-mediated lung tumorigenesis. **a** Immunohistochemistry analysis of pIGF-1R expression in wild-type (WT) and Agt^+/−^ mice treated with NNK. Representative images are shown in Supplementary Fig. 5c. **b–f** Western blot (WB) analyses of the indicated protein expression in whole cell lysates or conditioned medium (CM) from BEAS-2B cells that were untransfected (**b**–**e**) or transfected with GFP-IGF2 (**f**) or pretreated with losartan (Losa, 10 μM) (**c**), BAPTA-AM (BAP, 5 μM) (**f**), nimodipine (Nimo, 10 μM) (**f**), diazoxide (Dia, 20 μM) (**f**), or Exo1 (100 μM)(**f**) for 3 h and then stimulated with the indicated concentrations (**b**, **e**) or 0.1 μM (**c**, **d**, **f**) of angiotensin II (AngII) for 12 h (**b**, **c**, **e**, **f**) at the indicated times (**d**). **g** A time-lapse imaging analysis of BEAS-2B/GFP-IGF2 cells pretreated with BAPTA-AM (BAP, 5 μM) or Exo1 (Exo, 100 μM) for 3 h and then stimulated with AngII (0.1 μM) for 1 h. Arrows indicate secreted GFP-IGF2. Scale bar: 20 μm. **h** Confocal images of IGF2 (green), FM1-43 (red), and DAPI (blue) in BEAS-2B cells pretreated with BAPTA-AM (BAP, 5 μM) or Exo1 (Exo, 100 μM) for 3 h and then stimulated with AngII (0.1 μM) for 1 h. Arrows indicate perimembranous IGF2 within vesicles. Scale bar: 10 μm. **i** BEAS-2B cells (donor) were pretreated with BAPTA-AM (BAP, 5 μM) or Exo1 (Exo, 100 μM) for 3 h and stimulated with AngII (0.1 μM) for 12 h. The CM from the donor cells was added to recipient BEAS-2B cells for 30 min. Whole-cell lysates (WCL) from recipient cells were subjected to WB analysis. **j**, **k** Proliferation (**j**) and anchorage-dependent (AD) colony formation of BEAS-2B cells (**k**, left) and formation of foci of HBEL/p53i cells (**k**, right) treated with AngII (0.1 μM), either alone or together with linsitinib (Linsi, 0.1 μM) or an IGF2 neutralizing antibody (3 μg/mL) for 2 weeks. The bars represent the mean ± SD; *p < 0.05, **p < 0.01, and ***p < 0.001, as determined by one-way ANOVA with Dunnett’s post hoc test (**a, j, k**) or Brown-Forsythe and Welch ANOVA with Dunnett’s T3 post hoc test (**j**). Veh: vehicle.

We next investigated how NNK-induced activation of the α7nAChR/Src/STAT3/AngII pathway promotes transformation of pulmonary epithelial cells. Previous studies have shown that AngII/AGTR signaling can induce transcriptional upregulation^36^ or transactivation of IGF-1R^9^, which plays a key role in cell transformation^37^. We then hypothesized that NNK-induced AGT expression and subsequent AngII/AGTR signaling activation cause transformation of pulmonary epithelial cells *via* the IGF-1R signaling pathway. Indeed, genomic (Supplementary Fig. 5a) or pharmacological (Supplementary Fig. 5b) blockade of NNK-induced AngII/AGTR signaling activation [e.g., siRNA-mediated silencing of Src, STAT3, or AGT or treatment with dasatinib, Stattic, captopril, or losartan] inhibited NNK-induced IGF-1R phosphorylation in BEAS-2B cells. Moreover, administration of NNK increased the level of IGF-1R phosphorylation in the lungs of WT mice, but this effect was significantly abrogated in those of Agt^+/−^ mice (Fig. 5a, Supplementary Fig. 5c). These results suggest that the nAchR/Src/STAT3-mediated activation of the AngII/AGTR1 pathway plays an important role in NNK-mediated IGF-1R activation. Because IGF2 also transduces signals *via* IGF-2R^10^, we investigated whether IGF-2R was involved in the NNK-induced transformed phenotypes of lung epithelial cells. To this end, we established BEAS-2B cells in which IGF2R expression was knocked down by stable shRNA expression and then determined their response to NNK exposure. We observed that ablation of IGF2R expression moderately attenuated the effects of NNK exposure on anchorage-dependent colony formation, although the difference was not significant (Supplementary Fig. 5d). These findings suggest the possible involvement of IGF-2R in NNK-induced lung tumorigenesis.

We investigated the mechanism underlying NNK/AngII-induced IGF-1R activation in lung epithelial cells. We previously demonstrated NNK-mediated *IGF2* transcription through activation of STAT3^38^. Consistently, BEAS-2B cells exposed to NNK showed transcriptional upregulation of *IGF2*, which was suppressed by treatment with Stattic (Supplementary Fig. 5e). Thus, NNK-induced IGF-1R signaling activation in lung epithelial cells seemed to be attributable to STAT3-mediated *IGF2* transcription. We also examined the direct effect of AngII on IGF-1R phosphorylation. Exposure to AngII induced a dose-dependent activation of IGF-1R signaling in BEAS-2B cells at doses as low as 1 nM (Fig. 5b), which is achievable in human populations^39^, and this effect was suppressed by treatment with losartan (Fig. 5c). Notably, AngII rapidly activated IGF-1R as early as 5 min after exposure without affecting IGF-1R expression (Fig. 5d), indicating the presence of transcription-independent IGF-1R activation by AngII stimulation. Further analysis of the CM from AngII-treated cells revealed a dose-dependent increase in IGF2 but not IGF1 (Fig. 5e). We have previously shown that intracellular Ca^2+^ stimulates IGF2 secretion through the regulated secretory pathway^15^. Since the AngII/AGTR1 pathway is known to activate Gq, which mediates PLC-mediated calcium release^7^, we hypothesized that the AngII/AGTR1 signaling pathway activates the IGF-1R pathway through calcium-mediated IGF2 secretion. Pretreatment with a Ca^2+^ chelator (BAPTA-AM) or exocytosis inhibitors (nimodipine, diazoxide, or Exo1) decreased AngII-induced IGF2 secretion and IGF-1R activation (Fig. 5f). Live-cell time-lapse imaging analysis of BEAS-2B cells stably transfected with a GFP-conjugated IGF2 expression vector (BEAS-2B/GFP-IGF2 cells) revealed that exposure to AngII induced the secretion of GFP-IGF2 as early as 5 min, and treatment with BAPTA-AM or Exo1 markedly suppressed AngII-induced GFP-IGF2 secretion (Fig. 5g). Confocal microscopy with FM-1-43 dye, a fluorescent dye that labels exocytic vesicles^40^, clearly showed that AngII caused IGF2 exocytosis from BEAS-2B cells, which was completely blocked by BAPTA-AM or Exo1 treatment (Fig. 5h). Moreover, CM from the AngII-stimulated BEAS-2B cells (donor) induced IGF-1R phosphorylation in naïve BEAS-2B (recipient) cells (Fig. 5i). In contrast, CM from the AngII-stimulated donor cells that had been pretreated with BAPTA-AM or Exo1 failed to induce IGF-1R phosphorylation in recipient BEAS-2B cells (Fig. 5i). The capacity of AngII to stimulate proliferation (Fig. 5j) and colony and foci formation (Fig. 5k) was also significantly suppressed by treatment with linsitinib, an IGF-1R tyrosine kinase inhibitor, or an IGF2 neutralizing antibody. These results suggest that AngII-mediated IGF2 secretion and subsequent IGF-1R activation play an important role in NNK-induced acquisition of transformed phenotypes in lung epithelial cells.

Based on the reciprocal interaction among tumor cells, macrophages, and fibroblasts in tumorigenesis^41^, we further investigated the association of the interaction with macrophages and fibroblasts with NNK-induced lung tumorigenesis. IHC analysis of lung tissues revealed increases in arginase 1 (Arg1), a marker of immunosuppressive tumor-associated macrophages^42^, and α-SMA, a marker of cancer-associated fibroblasts^43^, in lung tissues from the NNK-exposed mice, while these NNK-induced increases were significantly attenuated in the Agt^+/−^ mice (Supplementary Fig. 6a). These results indicate that the NNK-induced AGT expression and subsequent AngII/AGTR1 signaling play a role in increases in macrophages and fibroblasts with protumor phenotypes. Because IGF2 secreted from cancer cells may stimulate surrounding macrophages and fibroblasts in a paracrine manner^44,45^, we next assessed the effect of CM from NNK-stimulated BEAS-2B cells on THP-1 and Wi38 cells. Exposure to CM from the NNK-treated BEAS-2B cells induced transcriptional increases in *CD206* and *CD163* in THP-1 cells (Supplementary Fig. 6b, left) and *ACTA2*, *COL1A1*, and *S100A4* in Wi38 fibroblasts (Supplementary Fig. 6b, right), but these effects were suppressed by linsitinib treatment. Hence, IGF2 secreted by Ca^2+^ release *via* AngII/AGTR1 signaling activation appears to induce IGF-1R signaling-mediated acquisition of M2- and CAF-associated phenotypes in macrophages and fibroblasts, respectively. These overall results suggest that activation of the AngII/AGTR1 pathway induces activation of IGF-1R signaling in pulmonary epithelial cells and stromal cells through increased production of IGF2, stimulating protumorigenic activities of these cells.

### Blockade of the lung RA system prevents NNK-induced lung tumor development

**Fig. 6:**
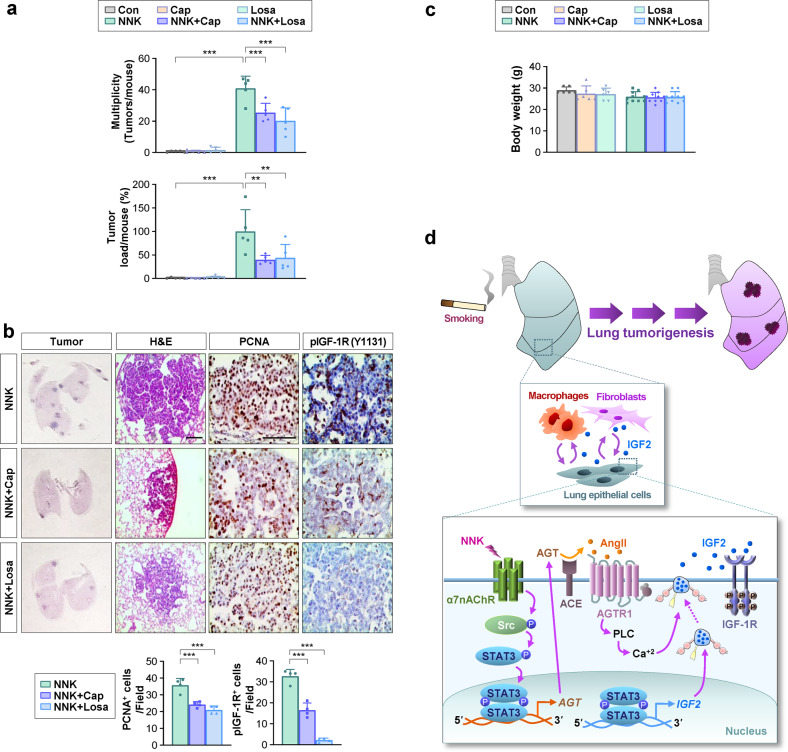
Pharmacological blockade of the AngII/AGTR1 pathway inhibits NNK-induced lung tumorigenesis. **a–c** Mice were treated with NNK (oral gavage, 3 μmol) in the absence or presence of losartan (Losa, oral gavage, 25 mg/kg) or captopril (Cap, oral gavage, 5 mg/kg). **a** Microscopic analyses of H&E-stained lung tissues for lung tumor formation (*n* ≥ 5). **b** H&E staining of lung tissues and immunohistochemical analyses of the indicated proteins in the lungs from the mice. Scale bars: 100 μm (H&E images), 50 μm (IHC images). **c** Body weight of mice at the end of the experiments [Con, Cap, Losa: *n* = 6; NNK, NNK + Cap, NNK + Losa: *n* = 10]. **d** Schematic model of lung tumorigenesis caused by NNK-induced changes in the pulmonary renin-angiotensin system. In light of our findings, NNK-induced activation of nAChR/Src/STAT3 signaling leads to transcriptional upregulation of AGT and IGF2. Increased AngII by the activity of ACE elevates protumorigenic activities in pulmonary epithelial cells and stromal cells through AGTR1-intervened calcium release and enhanced IGF2 secretion followed by the activation of the IGF-1R signaling pathway, thereby promoting lung tumor formation. The bars represent the mean ± SD; *p < 0.05, **p < 0.01, and ***p < 0.001, as determined by one-way ANOVA with Dunnett’s post hoc test (**a**, **b**) or Kruskal‒Wallis test with Dunn’s post hoc test (**a**). Con: control.

We investigated the effect of the AChR/Src/STAT3/AGT/AGTR1/IGF-1R signaling axis on NNK-induced lung tumorigenesis. Given the potential toxicity of targeting nAChR or Src^46,47^, the inhibition of the RA system by ACE inhibitors or AGTR1 antagonists that are prescribed to hypertensive patients without major complications would be an effective strategy for the prevention of lung cancer in smokers. Indeed, administration of captopril or losartan effectively inhibited tumor multiplicity and load (Fig. 6a). In addition, treatment with captopril or losartan, as well as treatment with methyllycaconitine and dasatinib, inhibited the expression of proliferating cell nuclear antigen (PCNA), a marker of cell proliferation^48^, and phosphorylated IGF-1R in the lungs of NNK-treated mice (Fig. 6b, Supplementary Fig. 7). No signs of toxicity, including significant changes in body weight, were observed during treatment (Fig. 6c). These findings support our hypothesis that the inhibition of the RA system by ACE inhibitors or AGTR1 antagonists can suppress TS-induced IGF-1R activation and lung tumorigenesis.

## Discussion

In this study, we show a novel mechanism whereby NNK-mediated control of the pulmonary RA system stimulates activation of the IGF-1R signaling pathway, contributing to TS-mediated lung tumorigenesis. Our study utilizing various pharmacological and genomic approaches targeting the nAChR/Src/STAT3 signaling cascade and mouse models carrying a lung-specific *Src* transgene or heterozygous *Agt* knockout demonstrated that the NNK-mediated control of the lung RA system is strictly through nAChR/Src/STAT3-mediated *AGT* transcription. Subsequent activation of the AngII/AGTR1 pathway appeared to promote lung tumorigenesis through the transformation of lung epithelial cells and protumoral polarization of fibroblasts and macrophages. Mechanistically, the AngII/AGTR1 pathway leads to increased production of IGF2 through STAT3-dependent transcriptional upregulation and Ca^2+^-dependent increased secretion, resulting in IGF-1R signaling activation (Fig. 6d). Finally, we show that treatment with clinically available RA system antagonists, including ACE inhibitors and ARBs, reduced NNK-induced lung tumorigenesis in mice. These results suggest that the lung RA system plays a pivotal role in TS-mediated lung tumor development, providing clinically available strategies for the prevention of TS-associated lung cancer.

Studies have shown that TS causes lung cancer development by inducing genetic/epigenetic alterations in oncogenes and tumor suppressor genes and by activating tumor-promoting signaling pathways^3–5^. Our analyses of public data from populations of smokers have identified overexpression of AGT in lung epithelial cells as an early biochemical event in human lung carcinogenesis. A significant correlation between lung epithelial AGT expression and poor prognosis of lung cancer patients was also observed. Our subsequent in vitro and in vivo experimental results included the following: (1) exposure to NNK induced transcriptional upregulation of *AGT* and secretion of AngII in lung epithelial cells, leading to the acquisition of transformed phenotypes in lung epithelial cells and protumoral capacities in macrophages and fibroblasts; (2) siRNA-mediated silencing of AGTR1 ablated NNK-mediated transformed phenotypes in lung epithelial cells; and (3) heterozygous *Agt* knockout suppressed the development of NNK-induced lung tumor development in mice, suggesting that the NNK-mediated activation of the AngII/AGTR1 pathway through modulation of AGT expression leads to the transformation of lung epithelial cells and the acquisition of a tumor-prone microenvironment, causing lung tumor development. We also observed NNK-induced upregulation of AGT in macrophages. Therefore, the regulation of AGT in macrophages and its role in NNK-induced lung tumorigenesis also need to be investigated in further studies.

The next question is how NNK stimulates the AngII/AGTR1 pathway. Our studies revealed a mechanism in which NNK binding to nAChRs stimulates Src-mediated activation of STAT3, a transcription factor for AGT expression^35^. Src, one of the nine Src family members, transduces signals between cell surface proteins, other intracellular proteins, and transcription factors^49^. Src can be activated by a myriad of stimuli that activate various transmembrane proteins, including adhesion receptors, receptor tyrosine kinases, G-protein coupled receptors (GPCRs) and cytokine receptors^50^. Aberrant Src activation has been implicated in the development of numerous human cancers, including lung cancer^51^. However, to date, the influence of Src on the lung RA system has not yet been elucidated. A previous report demonstrated that nicotine induces the activation of Src *via* a direct association between nAChR and β-arrestin^27^. Therefore, the NNK-induced activation of Src could be mediated *via* the NNK-mediated interaction of nAChR and β-arrestin. We found that NNK binding to nAChR induces activation of Src/STAT3, resulting in a transcription-dependent increase in *AGT* expression. Additionally, mice with lung-specific overexpression of Src exhibited AGT expression and tumor development in the lung, while heterozygous *Agt* knockout significantly suppressed Src-mediated lung tumor development. Therefore, Src appears to couple NNK/nAChR signaling to the AngII/AGTR1 pathway, thereby promoting pulmonary tumorigenesis.

Another important question is how the NNK/nAChR/AngII/AGTR1 signaling cascades promote pulmonary tumorigenesis. AngII has been shown to activate numerous signaling molecules, including the JAK/STAT pathway, the Src kinase family, the growth factor receptor family, and cell adhesion proteins, including paxillin and focal adhesion kinase (FAK)^52^. Additionally, the adapter protein SHC, tyrosine phosphatase SHP2, and insulin receptor substance 1 (IRS1) are phosphorylated in response to AngII^53^. Our study provided evidence that NNK-induced activation of the AngII/AGTR1 pathway leads to stimulation of the IGF-1R signaling pathway, which plays an essential role in cell transformation^37^ and TS-mediated lung tumorigenesis in mice and humans^15^. Importantly, inactivation of IGF-1R by a tyrosine kinase inhibitor significantly abrogated the AngII-mediated transformed phenotypes in lung epithelial cells and protumoral activities in macrophages and fibroblasts. Hence, the IGF-1R pathway appears to be essential for AngII-mediated pulmonary tumorigenesis.

We further explored how IGF-1R could be activated. Consistent with our previous report^38^, our analysis clearly identified the role of STAT3-dependent transcriptional upregulation of *IGF2* after exposure to NNK. However, our results, showing rapid IGF-1R signaling activation within 5 min after AngII exposure, indicated the presence of transcription-independent mechanisms underlying AngII-induced IGF-1R activation. Studies have shown that NNK-induced nAChR activation results in an increase in Ca^2+^ influx through voltage-dependent calcium channels (VDCCs), thereby inducing secretagogue-induced IGF2 secretion^15^, and that AngII/AGTR1 signaling activates phospholipase C (PLC), leading to inositol phosphate production and Ca^2+^ release within seconds^52^. Hence, we postulated that the AngII/AGTR1 pathway activates the IGF-1R pathway by two independent but sequential mechanisms, e.g., Src/STAT3-mediated transcriptional upregulation and secretagogue-induced IGF2 secretion. Importantly, suppression of the NNK-mediated AngII/AGTR1 pathway *via* blockade of ACE or AGTR1 significantly inhibited NNK-induced autocrine and paracrine IGF2-IGF-1R signaling and protumorigenic activities in pulmonary epithelial cells, macrophages, and fibroblasts and lung tumorigenesis in mice, which was consistent with previous reports^15,45,54^. These findings provide clear evidence that NNK-mediated control of the lung RA system through nAChR/Src/STAT3-mediated AngII production results in epithelial cell transformation and lung tumor development. Moreover, in addition to IGF-1R, IGF2 transduces signals *via* IGF-2R^10^, and a recent study demonstrated the association of IGF2/IGF-2R signaling with acquiring anti-inflammatory phenotypes in macrophages^55^. We also observed weak involvement of IGF-2R signaling in NNK-induced lung epithelial cell transformation. Thus, additional studies are necessary to investigate the precise role of IGF2/IGF-2R-mediated signaling in NNK/AngII pathway-mediated lung tumorigenesis.

Deregulation of the RA system has been implicated in pulmonary hypertension and inflammation, which mediates the onset of disorders associated with TS, such as chronic obstructive pulmonary disease (COPD)^8^. Epidemiological studies have shown that COPD is an independent risk factor for lung cancer^56,57^, and the use of ACE inhibitors or ARBs reduces the risks of COPD^58^ and lung cancer^59^. Although some contradictory results have also been reported^60^, which require further investigation, the results of our study demonstrate the potential of targeting the RA system as a novel target for the prevention of lung cancer associated with TS.

In summary, we identified a novel mechanism by which TC NNK induces sequential activation of the AngII/AGTR and IGF-1R signaling pathways in pulmonary epithelial cells and stromal cells to induce lung tumor development. We also show the potential clinical utility of ACE inhibitors and ARBs in suppressing NNK-mediated pathologic events. Our study has important clinical implications, shedding new light on the concept of repositioning ACE inhibitors and ARBs as a novel class of lung cancer chemopreventive agents without the potential deleterious toxicities found in metabolic disorders. Further preclinical and clinical studies are necessary to address this important point.

## Supplementary information

Supplementary information

## References

[1] Sung H (2021). Global Cancer Statistics 2020: GLOBOCAN estimates of incidence and mortality worldwide for 36 cancers in 185 countries. CA Cancer J. Clin..

[2] Hecht SS, Kassie F, Hatsukami DK (2009). Chemoprevention of lung carcinogenesis in addicted smokers and ex-smokers. Nat. Rev. Cancer.

[3] Hecht SS (1999). Tobacco smoke carcinogens and lung cancer. J. Natl. Cancer Inst..

[4] Schuller HM (2009). Is cancer triggered by altered signalling of nicotinic acetylcholine receptors?. Nat. Rev. Cancer.

[5] Schuller HM, Tithof PK, Williams M, Plummer H (1999). The tobacco-specific carcinogen 4-(methylnitrosamino)-1-(3-pyridyl)-1-butanone is a beta-adrenergic agonist and stimulates DNA synthesis in lung adenocarcinoma via beta-adrenergic receptor-mediated release of arachidonic acid. Cancer Res.

[6] Fyhrquist F, Saijonmaa O (2008). Renin-angiotensin system revisited. J. Intern. Med.

[7] George AJ, Thomas WG, Hannan RD (2010). The renin-angiotensin system and cancer: old dog, new tricks. Nat. Rev. Cancer.

[8] Shrikrishna D, Astin R, Kemp PR, Hopkinson NS (2012). Renin-angiotensin system blockade: a novel therapeutic approach in chronic obstructive pulmonary disease. Clin. Sci.

[9] Bouallegue A, Vardatsikos G, Srivastava AK (2009). Role of insulin-like growth factor 1 receptor and c-Src in endothelin-1- and angiotensin II-induced PKB phosphorylation, and hypertrophic and proliferative responses in vascular smooth muscle cells. Can. J. Physiol. Pharmacol..

[10] Pollak MN, Schernhammer ES, Hankinson SE (2004). Insulin-like growth factors and neoplasia. Nat. Rev. Cancer.

[11] Tamura M (2008). Specific single chain variable fragment (ScFv) antibodies to angiotensin II AT(2) receptor: evaluation of the angiotensin II receptor expression in normal and tumor-bearing mouse lung. J. Mol. Histol..

[12] Kanehira T (2005). Angiotensin II type 2 receptor gene deficiency attenuates susceptibility to tobacco-specific nitrosamine-induced lung tumorigenesis: involvement of transforming growth factor-beta-dependent cell growth attenuation. Cancer Res.

[13] Sato M (2006). Multiple oncogenic changes (K-RAS(V12), p53 knockdown, mutant EGFRs, p16 bypass, telomerase) are not sufficient to confer a full malignant phenotype on human bronchial epithelial cells. Cancer Res..

[14] Klein-Szanto AJ (1992). A tobacco-specific N-nitrosamine or cigarette smoke condensate causes neoplastic transformation of xenotransplanted human bronchial epithelial cells. Proc. Natl. Acad. Sci. USA.

[15] Boo HJ (2016). The tobacco-specific carcinogen-operated calcium channel promotes lung tumorigenesis via IGF2 exocytosis in lung epithelial cells. Nat. Commun..

[16] Sarzani R (2010). Angiotensinogen promoter variants influence gene expression in human kidney and visceral adipose tissue. J. Hum. Hypertens..

[17] Messeguer X (2002). PROMO: detection of known transcription regulatory elements using species-tailored searches. Bioinformatics.

[18] Cho, J. et al. RGS2-mediated translational control mediates cancer cell dormancy and tumor relapse. *J. Clin. Invest*. **131**, e136779 (2021). 10.1172/JCI136779 PMC7773398 33393490

[19] Livak KJ, Schmittgen TD (2001). Analysis of relative gene expression data using real-time quantitative PCR and the 2(T)(-Delta Delta C) method. Methods.

[20] Miller YE (2003). Induction of a high incidence of lung tumors in C57BL/6 mice with multiple ethyl carbamate injections. Cancer Lett..

[21] Min JH (2020). 3,4,5-Trihydroxycinnamic acid exerts a protective effect on pulmonary inflammation in an experimental animal model of COPD. Int. Immunopharmacol..

[22] Oga T (2009). Prostaglandin F(2alpha) receptor signaling facilitates bleomycin-induced pulmonary fibrosis independently of transforming growth factor-beta. Nat. Med..

[23] Goldstein B, Trivedi M, Speth RC (2017). Alterations in gene expression of components of the Renin-angiotensin system and its related enzymes in lung cancer. Lung Cancer Int.

[24] Weber SM (2011). Tobacco-specific carcinogen nitrosamine 4-(methylnitrosamino)-1-(3-pyridyl)-1-butanone induces AKT activation in head and neck epithelia. Int. J. Oncol..

[25] Chong G, Kuo FW, Tsai S, Lin C (2017). Validation of reference genes for cryopreservation studies with the gorgonian coral endosymbiont Symbiodinium. Sci. Rep..

[26] Byrne AJ, Mathie SA, Gregory LG, Lloyd CM (2015). Pulmonary macrophages: key players in the innate defence of the airways. Thorax..

[27] Dasgupta P (2006). Nicotine induces cell proliferation by beta-arrestin-mediated activation of Src and Rb-Raf-1 pathways. J. Clin. Invest..

[28] Garcia R (2001). Constitutive activation of Stat3 by the Src and JAK tyrosine kinases participates in growth regulation of human breast carcinoma cells. Oncogene.

[29] Westhoff MA, Serrels B, Fincham VJ, Frame MC, Carragher NO (2004). SRC-mediated phosphorylation of focal adhesion kinase couples actin and adhesion dynamics to survival signaling. Mol. Cell Biol..

[30] Malkinson AM (1998). Molecular comparison of human and mouse pulmonary adenocarcinomas. Exp. Lung Res..

[31] Wert SE, Glasser SW, Korfhagen TR, Whitsett JA (1993). Transcriptional elements from the human SP-C gene direct expression in the primordial respiratory epithelium of transgenic mice. Dev. Biol..

[32] Choi JH, Nguyen MP, Lee D, Oh GT, Lee YM (2014). Hypoxia-induced endothelial progenitor cell function is blunted in angiotensinogen knockout mice. Mol. Cells.

[33] Li J, Brasier AR (1996). Angiotensinogen gene activation by angiotensin II is mediated by the rel A (nuclear factor-kappaB p65) transcription factor: one mechanism for the renin angiotensin system positive feedback loop in hepatocytes. Mol. Endocrinol..

[34] Demura M, Demura Y, Takeda Y, Saijoh K (2015). Dynamic regulation of the angiotensinogen gene by DNA methylation, which is influenced by various stimuli experienced in daily life. Hypertens. Res..

[35] Ray S, Sherman CT, Lu M, Brasier AR (2002). Angiotensinogen gene expression is dependent on signal transducer and activator of transcription 3-mediated p300/cAMP response element binding protein-binding protein coactivator recruitment and histone acetyltransferase activity. Mol. Endocrinol..

[36] Jia G, Aggarwal A, Yohannes A, Gangahar DM, Agrawal DK (2011). Cross-talk between angiotensin II and IGF-1-induced connexin 43 expression in human saphenous vein smooth muscle cells. J. Cell Mol. Med..

[37] Zahradka P, Storie B, Wright B (2009). IGF-1 receptor transactivation mediates Src-dependent cortactin phosphorylation in response to angiotensin II. Can. J. Physiol. Pharmacol..

[38] Min HY (2016). Smoking-associated lung cancer prevention by blockade of the beta-adrenergic receptor-mediated insulin-like growth factor receptor activation. Oncotarget.

[39] Bloem LJ, Manatunga AK, Tewksbury DA, Pratt JH (1995). The serum angiotensinogen concentration and variants of the angiotensinogen gene in white and black children. J. Clin. Invest..

[40] Amaral E, Guatimosim S, Guatimosim C (2011). Using the fluorescent styryl dye FM1-43 to visualize synaptic vesicles exocytosis and endocytosis in motor nerve terminals. Methods Mol. Biol.

[41] Buechler MB, Fu W, Turley SJ (2021). Fibroblast-macrophage reciprocal interactions in health, fibrosis, and cancer. Immunity.

[42] Arlauckas SP (2018). Arg1 expression defines immunosuppressive subsets of tumor-associated macrophages. Theranostics.

[43] Han C, Liu T, Yin R (2020). Biomarkers for cancer-associated fibroblasts. Biomark. Res..

[44] Lee JS (2015). STAT3-mediated IGF-2 secretion in the tumour microenvironment elicits innate resistance to anti-IGF-1R antibody. Nat. Commun..

[45] Xu WW (2017). Cancer cell-secreted IGF2 instigates fibroblasts and bone marrow-derived vascular progenitor cells to promote cancer progression. Nat. Commun..

[46] Egleton RD, Brown KC, Dasgupta P (2008). Nicotinic acetylcholine receptors in cancer: multiple roles in proliferation and inhibition of apoptosis. Trends Pharmacol. Sci..

[47] Conchon M, Freitas CM, Rego MA, Braga Junior JW (2011). Dasatinib—clinical trials and management of adverse events in imatinib resistant/intolerant chronic myeloid leukemia. Rev. Bras. Hematol. Hemoter..

[48] Kubben FJ (1994). Proliferating cell nuclear antigen (PCNA): a new marker to study human colonic cell proliferation. Gut.

[49] Bjorge JD, Jakymiw A, Fujita DJ (2000). Selected glimpses into the activation and function of Src kinase. Oncogene.

[50] Zhang S, Yu D (2012). Targeting Src family kinases in anti-cancer therapies: turning promise into triumph. Trends Pharmacol. Sci..

[51] Irby RB, Yeatman TJ (2000). Role of Src expression and activation in human cancer. Oncogene.

[52] Forrester SJ (2018). Angiotensin II signal transduction: an update on mechanisms of physiology and pathophysiology. Physiol. Rev..

[53] Guo DF, Sun YL, Hamet P, Inagami T (2001). The angiotensin II type 1 receptor and receptor-associated proteins. Cell Res..

[54] Du L (2019). IGF-2 preprograms maturing macrophages to acquire oxidative phosphorylation-dependent anti-inflammatory properties. Cell Metab.

[55] Wang, X. et al. IGF2R-initiated proton rechanneling dictates an anti-inflammatory property in macrophages. *Sci. Adv*. **6**, eabb7389 (2020). 10.1126/sciadv.abb7389 PMC7688333 33239287

[56] Ahn SV (2020). Cancer development in patients with COPD: a retrospective analysis of the National Health Insurance Service-National Sample Cohort in Korea. BMC Pulm. Med..

[57] Spyratos D, Papadaki E, Lampaki S, Kontakiotis T (2017). Chronic obstructive pulmonary disease in patients with lung cancer: prevalence, impact and management challenges. Lung Cancer.

[58] Vasileiadis IE, Goudis CA, Giannakopoulou PT, Liu T (2018). Angiotensin converting enzyme inhibitors and angiotensin receptor blockers: a promising medication for chronic obstructive pulmonary disease?. COPD.

[59] Wang KL (2013). Long-term use of angiotensin II receptor blockers and risk of cancer: a population-based cohort analysis. Int. J. Cardiol..

[60] Sipahi I, Debanne SM, Rowland DY, Simon DI, Fang JC (2010). Angiotensin-receptor blockade and risk of cancer: meta-analysis of randomised controlled trials. Lancet Oncol..

