# Supplementary material for: The tobacco-specific carcinogen NNK induces pulmonary tumorigenesis via nAChR/Src/STAT3-mediated activation of the renin-angiotensin system and IGF-1R signaling
Source: Exp Mol Med. 2023 Jun 1;55(6):1131–44. doi: 10.1038/s12276-023-00994-2 (PMC10317988; doi:10.1038/s12276-023-00994-2)
Supplement: Supplementary file 1 — Supplementary information [file 12276_2023_994_MOESM1_ESM.pdf]

Supplementary materials

The tobacco-specific carcinogen NNK induces pulmonary tumorigenesis via nAChR/Src/STAT3-mediated activation of the renin-angiotensin system and IGF-1R signaling

Supplementary Table 1. A list of probes that were used to extract gene expression levels from GEO datasets.

Gene	Probe
REN	206367_at
AGT	202834_at
AGTR1	208016_s_at
AGTR2	207293_s_at
MAS1	208210_at
ACE	209749_s_at
ENPEP	204845_s_at
MME	203435_s_at
PRCP	1569205_at
SRC	1565080_at
CEBPB	212501_at
STAT3	208991_at
NR3C1	216321_s_at
NR3C2	205259_at
RELA	209878_s_at

Supplementary Table 2. Primer sequences used in this study.

Gene	Species	Forward sequence (5´-3´)	Reverse sequence (5´-3´)	Application
AGT	Human	GCACCTCAGTGTCTGTTCCCAT	ACCGAGAAGTTGTCCTGGATGT	Real-time PCR
AGT	Human	TCTCCCCGGACCATCCA	TGCTCAATTTTTGCAGGTTCAG	Real-time PCR
IGF2	Human	GCGGCTTCTACTTCAGCAG	CAGGTGTCATATTGGAACAAC	Real-time PCR
CD163	Human	AGTCTGCTCAAGATACACAGA AA	GGTAGAAAGGGCAACTCCACA	Real-time PCR
CD206	Human	CCATCGAGGAAGAGGTTCGG	GGTGGGTTACTCCTTCTGCC	Real-time PCR
ACTA2	Human	CGTTACTACTGCTGAGCGTGA	GCCCATCAGGCAACTCGTAA	Real-time PCR
COL1A1	Human	CCTGGAAAGAATGGAGATGATG	ATCCAAACCACTGAAACCTCTG	Real-time PCR
S100A4	Human	AGGGTGACAAGTTCAAGCTCAA	GCAGGACAGGAAGACACAGTA	Real-time PCR
ACTB	Human	ACTACCTCATGAAGATC	GATCCACATCTGCTGGAA	Real-time PCR
ACTB	Human	GCGAGAAGATGACCCAGATC	GGATAGCACAGCCTGGATAG	Real-time PCR
RN18S	Human	GCAATTATTCCCCATGAACG	GGCCTCACTAAACCATCCAA	Real-time PCR
AGT	Human	CCCTGGCTTTCAACACCTAC	CTGTGGGCTCTCTCTCATCC	RT-PCR
ACTB	Human	ACTACCTCATGAAGATC	GATCCACACATCTGCTGGAA	RT-PCR
Agt	Mouse	ACAGCATCTCGGTGTCTGTG	TGTCGAGATCTGAGGTGCAG	Real-time PCR
Ace	Mouse	CACTATGGGTCCGAGTACAT	ATCATAGATGTTGGACCAGG	Real-time PCR
Prcp	Mouse	GAACTACCCTTACGCATGCAACT	AATATTGGCACACCTCCTTGATG	Real-time PCR
Mme	Mouse	CAGCCTCAGCCGAAACTACA	TTTGTCTCAGCATCCATCCAA	Real-time PCR
Agtr1a	Mouse	AGAACACCAATATCACTGTTTG	TAGCTGGTAAGAATGATTAGGA	Real-time PCR
Agtr1b	Mouse	CTGCTATGCCCATCACCATCTG	GATAACCCTGCATGCGACCTG	Real-time PCR
Agtr2	Mouse	GTGCATGCGGGAGCTGAGTA	ATTGGTGCCAGTTGCGTTGA	Real-time PCR
Ren	Mouse	ATGAAGGGGGTGTCTGTGGGGTC	ATGTCGGGGAGGGTGGGCACCTG	Real-time PCR
Rn18s	Mouse	GGAATAATGGAATAGGACCG	TCTGTCAATCCTGTCCGTGTCC	Real-time PCR

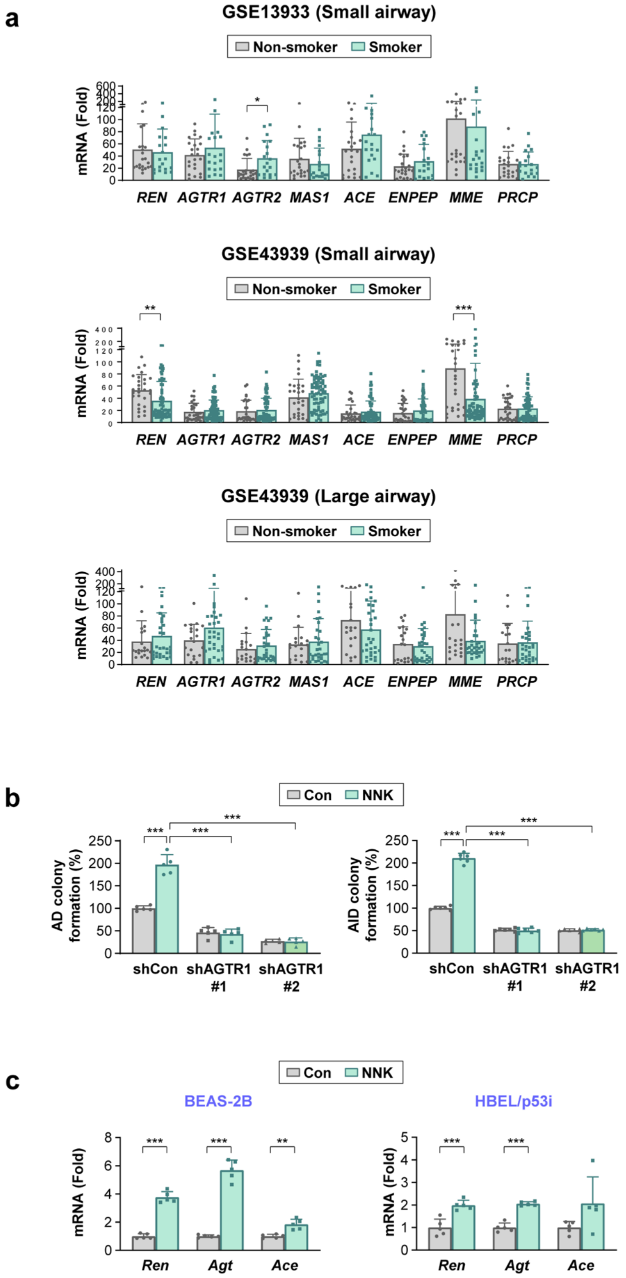

Supplementary Fig. 1. Regulation of components of the renin-angiotensin system in smokers or by NNK treatment and association of AGTR1 with NNK-induced lung epithelial cell transformation. (**a**) Analysis of publicly available datasets for the expression of RA system components in the airway epithelium of smokers [GSE13933: n = 19; GSE43939 (small airway): n = 69; GSE43939 (large airway): n = 31] vs. non-smokers [GSE13933: n = 23; GSE43939 (small airway): n = 28; GSE43939 (large airway): n = 20]. (**b**) Changes in the NNK-induced anchorage-dependent (AD) and -independent (AID) colony formation by knockdown of AGTR1 expression in 1170 cells. (**c**) Real-time PCR analysis for the regulation of *Ren*, *Agt*, and *Ace* expression in two lung epithelial cells (BEAS-2B and HBEL/p53i cells) by treatment with NNK (10 μM) for 24 h. The bars represent the mean ± SD; **$p < 0.01$, and ***$p < 0.001$, as determined by Mann-Whitney test (**a**), one-way ANOVA with Dunnett's post-hoc test (**b**), or a two-tailed Student's t-test (**c**) by comparison with the indicated group.

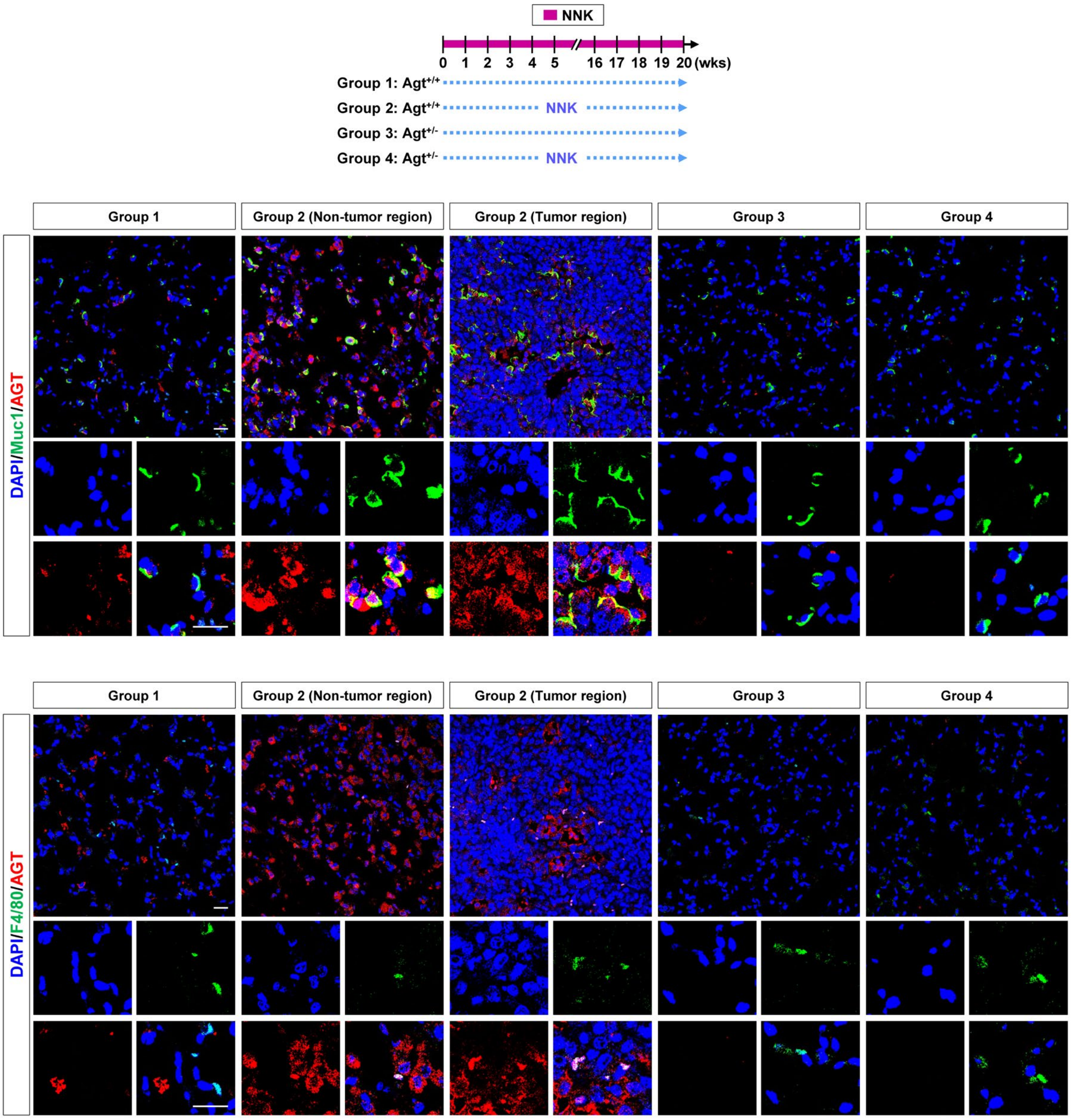

Supplementary Fig. 2. Changes in AGT expression in alveolar type 2 epithelial cells and macrophages of the lungs by NNK treatment. Immunofluorescence analysis for the AGT expression in mucin1-positive (Muc1^{+}) alveolar type 2 epithelial cells and F4/80^{+} macrophages. Scale bars: 20 μm.

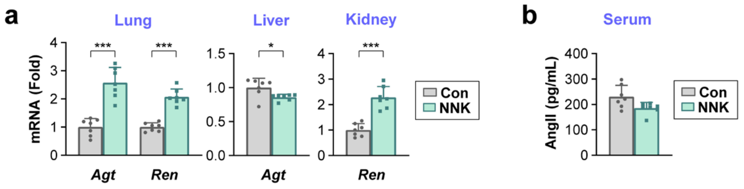

Supplementary Fig. 3. Changes in AGT and renin expression in the lung, liver, kidney, and blood by NNK treatment. (**a**, **b**) Real-time PCR (**a**) and ELISA (**b**) for the determination of the expression of angiotensinogen (AGT) and renin in the lung, liver, or kidney of NNK-treated mice (**a**) and the level of angiotensin II (AngII) in the serum of NNK-treated mice (**b**). The bars represent the mean ± SD; *$p < 0.05$, **$p < 0.01$, and ***$p < 0.001$, as determined by a two-tailed Student's t-test by comparison with the indicated group.

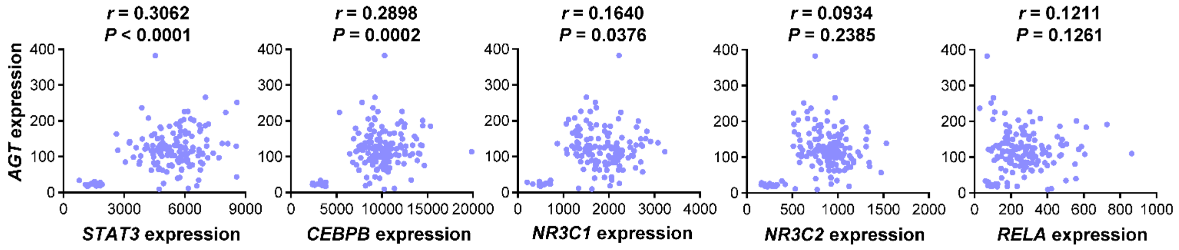

Supplementary Fig. 4. The correlation of AGT expression with expression of several transcription factors. Analysis of the GSE18385 dataset (*n* = 161) for the Spearman rank correlation between *AGT* mRNA expression and mRNA expression of several transcription factors such as *STAT3*, *CEBPB*, *NR3C1*, *NR3C2*, and *RELA*.

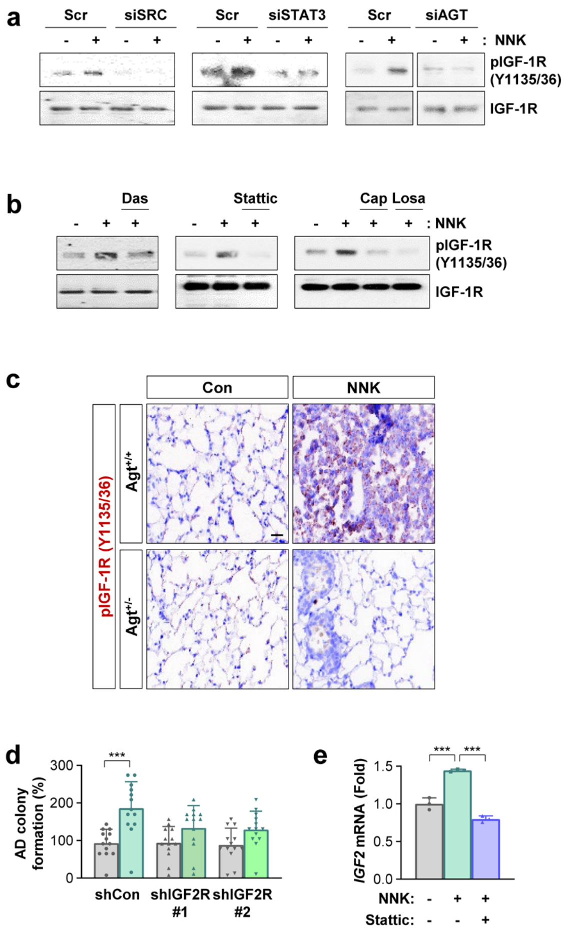

Supplementary Fig. 5. Regulation of NNK-induced IGF-1R phosphorylation and IGF2 transcription by blockade of Src, STAT3, angiotensin II, ACE, and AGTR1. (**a, b**) Western blot (WB) analysis for pIGF-1R and IGF-1R expression in BEAS-2B cells treated with NNK (10 μM), either alone or together with siRNA-mediated silencing of SRC, STAT3, or AGT (**a**) or pharmacological blockade of Src, STAT3, ACE, or AGTR1 by treatment with dasatinib (Das, 0.1 μM), Stattic (1 μM), captopril (Cap, 1 μM), or losartan (Losa, 10 μM) for 24 h (**b**). (**c**) Representative immunohistochemistry images for regulation of NNK-induced IGF-1R phosphorylation in the lungs of $Agt^{+/+}$ and $Agt^{+/-}$ mice (n = 6/group). Scale bar: 100 μm. Quantitative analysis results are shown in **Fig. 5a**. (**d**) The anchorage-dependent (AD) colony formation assay for determining changes in AD colony formation by knockdown of IGF2R expression in BEAS-2B cells. (**e**) Real-time PCR analysis for regulation of IGF2 expression in BEAS-2B cells stimulated with NNK (10 μM) in the absence or presence of Stattic (1 μM). The bars represent the mean ± SD; ***p < 0.001, as determined by one-way ANOVA with Tukey's post-hoc test.

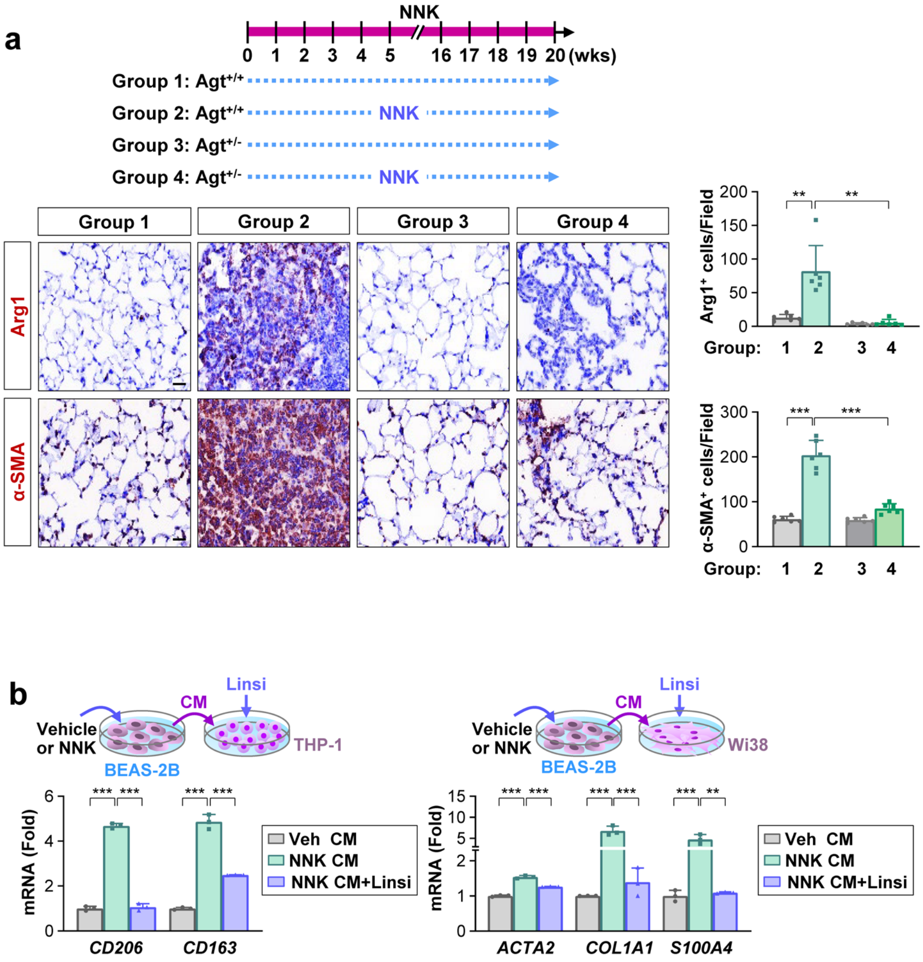

Supplementary Fig. 6. NNK/AGT/AngII/AGTR1-mediated IGF-1R signaling modulates recruitment and phenotypes of macrophages and fibroblasts, promoting NNK-mediated lung tumorigenesis. (**a**) Immunohistochemistry analysis of arginase 1 (Arg1) and α-smooth muscle actin (α-SMA) expression in wild-type (WT, $Agt^{+/+}$) and $Agt^{+/-}$ mice treated with NNK (n = 6/group). Scale bars: 100 μm. (**b**) Real-time PCR analysis of *CD206* and *CD163* expression in THP-1 cells and *ACTA2*, *COL1A1*, and *S100A4* expression in Wi38 fibroblasts. BEAS-2B cells (donor) were treated with NNK (10 μM) for 1 day. THP-1 and Wi38 cells pretreated with linsitinib (Linsi, 0.1 μM) for 3 h were stimulated with CM from donor cells for 1 day. The bars represent the mean ± SD; *$p < 0.05$, **$p < 0.01$, and ***$p < 0.001$, as determined by one-way ANOVA with Dunnett's post-hoc test (**a, b**) or Kruskal–Wallis test with Dunn's post hoc test (**a**).

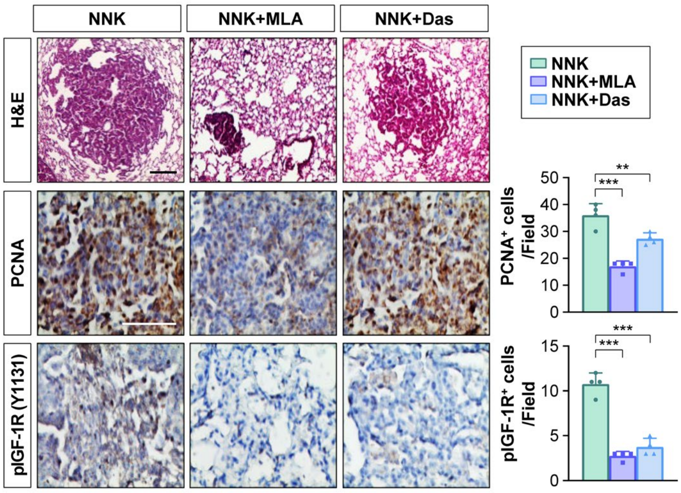

Supplementary Fig. 7. Regulation of PCNA expression and NNK-induced IGF-1R phosphorylation by treatment with methyllycaconitine and dasatinib. Representative immunohistochemistry images for regulation of proliferating cell nuclear antigen (PCNA) and NNK-induced Src phosphorylation and AGT expression in the lungs of mice treated with NNK (oral gavage, 3 μmol), either alone or in combination with dasatinib (Das, oral gavage, 10 mg/kg) or methyllycaconitine (MLA, oral gavage, 1 mg/kg).
